# Recent Advances and Perspectives on Coupled Water Electrolysis for Energy‐Saving Hydrogen Production

**DOI:** 10.1002/advs.202411964

**Published:** 2025-01-07

**Authors:** Jiachen Li, Yuqiang Ma, Xiaogang Mu, Xuanjun Wang, Yang Li, Haixia Ma, Zhengxiao Guo

**Affiliations:** ^1^ Department of Chemistry The University of Hong Kong Hong Kong 999077 China; ^2^ Xi'an Key Laboratory of Special Energy Materials, School of Chemical Engineering Northwest University Xi'an 710069 China; ^3^ Zhijian Laboratory Xi'an 710025 China; ^4^ Shaanxi Key Laboratory of Degradable Biomedical Materials School of Chemical Engineering Northwest University Xi'an 710069 China

**Keywords:** coupled water electrolysis, electrocatalysis, hydrogen production, industrial‐scale current density, small molecule oxidation

## Abstract

Overall water splitting (OWS) to produce hydrogen has attracted large attention in recent years due to its ecological‐friendliness and sustainability. However, the efficiency of OWS has been forced by the sluggish kinetics of the four‐electron oxygen evolution reaction (OER). The replacement of OER by alternative electrooxidation of small molecules with more thermodynamically favorable potentials may fundamentally break the limitation and achieve hydrogen production with low energy consumption, which may also be accompanied by the production of more value‐added chemicals than oxygen or by electrochemical degradation of pollutants. This review critically assesses the latest discoveries in the coupled electrooxidation of various small molecules with OWS, including alcohols, aldehydes, amides, urea, hydrazine, etc. Emphasis is placed on the corresponding electrocatalyst design and related reaction mechanisms (e.g., dual hydrogenation and N–N bond breaking of hydrazine and C═N bond regulation in urea splitting to inhibit hazardous NCO^−^ and NO^−^ productions, etc.), along with emerging alternative electrooxidation reactions (electrooxidation of tetrazoles, furazans, iodide, quinolines, ascorbic acid, sterol, trimethylamine, etc.). Some new decoupled electrolysis and self‐powered systems are also discussed in detail. Finally, the potential challenges and prospects of coupled water electrolysis systems are highlighted to aid future research directions.

## Graphical abstract

Replacing oxygen evolution reaction (OER) with thermodynamically favorable small molecules oxidation reactions to construct coupled water electrolysis systems has been extensively investigated in the last decade. This review focuses on the fundamental principle and development of coupled system, the electrocatalysts design, and proposed mechanism for small molecules oxidation reactions. Perspectives and challenges are highlighted for future advancement in this field.

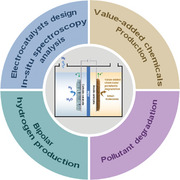

## 1 Introduction

Clean hydrogen (H_2_) obtained from electrochemical or photoelectrochemical water splitting is considered as innovative technologies for future energy resource.^[^ ^1^, ^2^ ^]^ Such a sustainable strategy enables global zero carbon emission and avoids serious environmental contamination brought by the nonrenewable fossil fuels (coal, oil, and natural gas).^[^ ^3^, ^4^, ^5^, ^6^ ^]^ The electrochemical overall water splitting (OWS, H_2_O (l) → H_2_ (g) + 1/2 O_2_ (g), Δ*G*° = +237.2 kJ mol^−1^, Δ*E*° = 1.23 V) consists of two half‐cell reactions: hydrogen evolution reaction (HER) and oxygen evolution reaction (OER).^[^ ^4^, ^7^ ^]^ The OWS in alkaline medium is commonly studied because the activity of OER in alkaline is relatively higher than that acid medium, and prevents the consumption of expensive proton exchange membranes.^[^ ^8^, ^9^ ^]^ In alkaline reaction system, the HER involves two‐step process of water dissociation (Volmer step) to supply protons and then convert to H_2_ molecules, which proceed two different pathways of directly combining two protons to form H_2_ (Tafel step) or producing protons from Volmer step that combines with another water molecules to form H_2_ (Heyrovsky step).^[^ ^10^ ^]^ At the counter side, OER is a complicated and sluggish four‐electron transfer kinetic process, involving a diversity of oxygen‐containing intermediates with higher thermodynamic potential (1.23 V vs RHE) than that of HER (0 V vs RHE). In view of this, OER is considered as main energy consumption source (90% of the energy input) of the water electrolysis that leads to high cell voltage (e.g., >1.6 V) of OWS for achieving a significant producing of H_2_ in practice.^[^ ^11^, ^12^ ^]^ Designing nonprecious electrocatalysts is considered to be an efficient strategy to improve the OER performance. Nevertheless, the development of advanced non‐noble electrocatalysts for OER still delivers mediocre activity and stability. Undeniably, the design of highly efficient OER catalysts would only reduce the overpotential between thermodynamic potential (1.23 V vs RHE) and working potential, and cannot breakthrough the restriction of high thermodynamic potential of OER, which is not able to significantly reduce the cell voltage and energy consumption of OWS. In addition to the sluggish kinetic rate of OER, some other challenges should be noted. First, strictly coupled HER and OER would result in the formation of H_2_ and O_2_ simultaneously, which may result in potential explosive consequences when the H_2_/O_2_ across the membrane and mixes together. Specifically, H_2_ crossover may be enhanced by stack compression because of the reduced mass transfer that leads to H_2_ supersaturation. In addition, the high operating temperature and pressure (e.g., PEMWE, ≈60 °C, 130 bars) at a high current density may induce production of H_2_O_2_ and other reactive oxygen species (ROS) to attack the ionomer of membrane and cause chain scission, unzipping, ionomer degradation, and functional group destruction. This may result in membrane thinning, short circuits, generation of hot spots, and potential risk of flammable gas mixing.^[^ ^13^ ^]^ Second, the coexistence of H_2_/O_2_ forms reactive oxygen species (ROS) and shortens the lifetime of the membrane, electrode catalysts, and electrolyzer. Third, the produced O_2_ may diffuse to cathodic side through gas crossover and then reduced to H_2_O_2_ by oxygen reduction reaction (ORR) to consume extra energy input and degrade the membrane. Finally, O_2_ is less valuable than H_2_ fuel, hence, O_2_ is merely a side product during water electrolysis.^[^ ^14^, ^15^, ^16^ ^]^

**Figure 1:**
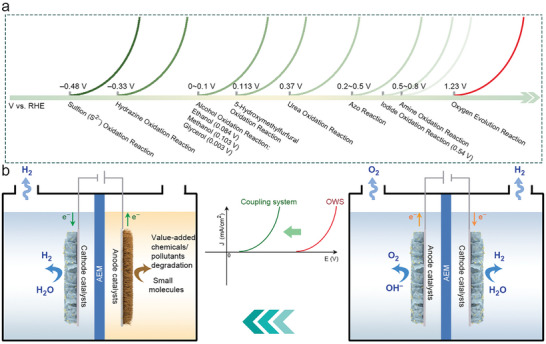
a) Graphic illustration of thermodynamic equilibrium potentials for various oxidation reactions. b) Schematic diagram of traditional electrolyzer and coupled system and corresponding polarization curves.

Given the above challenges brought by the OER, it is urgent to investigate new electrolyzer systems that can tackle the named challenges and realize H_2_ production with a lower energy input. In this review, we summarize the nonconventional electrolyzer systems starting with the conception of decoupled water electrolysis, and thereafter, we attach great importance to recent advances in coupled systems based on the various small molecules oxidation (hydrazine, urea, alcohols, aldehydes, amines, sulfides, etc.) with lower thermodynamic potential than OER (**Figure** **1**a,b). The corresponding electrocatalysts design for the systems is also presented. Apart from the advantages brought by the low thermodynamic potential of small molecules, the green electrooxidation synthesis of these small molecules to value‐added chemicals or pollutants degradation can avoid traditional hazardous condition. For example, the oxidative degradation of hydrazine pollutants by the electrochemical pathway can fast remove hydrazine from industrial sewage and avoid using extra oxidants (e.g., Fenton's reagent) or complex separation.^[^ ^17^ ^]^ The electrosynthesis of azo energetic compounds can well evade high‐temperature synthetic condition (≈100 °C), hazardous or corrosive reagents (KMnO_4_, (NH_4_)_2_S_2_O_8_, NaOCl/NaOBr, etc.) brought by traditional synthetic pathway.^[^ ^18^ ^]^ Therefore, the integration of small molecules electrooxidation and H_2_ production will achieve “kill two birds with one stone” aim for low‐energy‐consumption H_2_ production and obtain value‐added chemicals or pollutants degradation through an environmentally friendly protocol. A quantity of reviews regarding to the coupled systems of energy‐saving H_2_ production and various anodic reactions have been reported.^[^ ^11^, ^12^, ^15^, ^19^, ^20^, ^21^, ^22^ ^]^ It is still necessary to update the recent advances on the OWS coupled systems and new emerging alternative anodic reactions on the electrooxidation of small molecules, such as tetrazoles, furazans, iodide, quinolines, ascorbic acid, sterol, trimethylamine, etc. In addition, new proposed mechanisms of dual hydrogenation (200% Faradic efficiency for hydrogen production), N–N bond breakage mechanism of hydrazine, and C⎓N bond regulation in urea splitting to inhibit hazardous NCO^−^ and NO^−^ productions are presented to guide further design and development of highly‐efficient coupled water electrolysis. Finally, the potential challenges and some future development directions toward coupled H_2_ production systems are outlined.

*i) Origin of coupled water electrolysis*: In view of the drawbacks by OER in conventional OWS, the coupled water electrolysis is developed, following the concept of decoupled water electrolyzer system (DWE). Hence, a brief discussion of the DWE is firstly introduced before the review of coupled water electrolysis.

**Figure 2:**
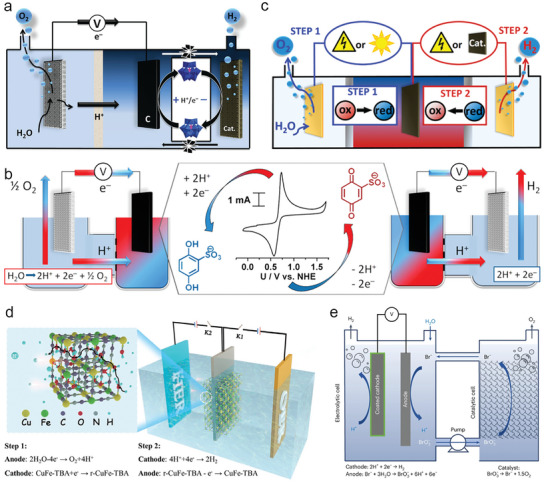
Schematic of decoupled water electrolysis system using a) H_4_[SiW_12_O_40_]. Reproduced with permission.^[^ ^25^ ^]^ Copyright 2014. American Association for the Advancement of Science. b) Small molecular weight quinone derivative. Reproduced with permission.^[^ ^26^ ^]^ Copyright 2013. American Chemistry Society. c) H_3_PMo_12_O_40_ as the electron‐coupled‐proton buffer (ECPB). Reproduced with permission.^[^ ^24^ ^]^ Copyright 2016. American Chemistry Society. d) Graphitc illustration of prussian blue analogs as a solid‐state redox mediator to decouple water electrolysis. Reproduced with permission.^[^ ^34^ ^]^ Copyright 2021. Wiley‐VCH. e) Schematic illustration of NaBr redox reaction as mediator to decouple water electrolysis. Reproduced with permission.^[^ ^38^ ^]^ Copyright 2024. Springer Nature.

*ii) Liquid‐state redox mediator‐based DWE*: Cronin et al.^[^ ^23^ ^]^ firstly propose the conception of DWE using LRM to decouple HER and OER. In their contributions, a new intermediates of electron‐coupled‐proton buffer (ECPB) is proposed using polyoxometalate H_3_PMo_12_O_40_ as a LRM, the OER can be taken up reversibly by ECPB for hydrogen production, realizing the separation of H_2_/O_2_ and avoid the potential explosion (Figure 2c).^[^ ^24^ ^]^ This new strategy can also obtain pure H_2_ without the additional gas purification process. Following this conception, their group introduced H_4_[SiW_12_O_40_] as the ECPB (**Figure** **2**a).^[^ ^25^ ^]^ However, the high molecular weight of such ECPB does not be at a practical advantage due to the limited buffering capacity built into the system and the temporal separation of H_2_ and O_2_. Based on these issues, they introduce a low molecular weight and more abundant elements of quinone derivative illuminated by natural photosynthetic systems as the LRM to successfully decouple H_2_ evolution from OER and lower the cost of quinone derivative ECPB (Figure 2b).^[^ ^26^ ^]^ As a result, the hydroquinone/benzoquinone LRM show an effective redox couple to decouple the OER from HER. This work extends the ECPB concept from inorganic to organic molecules as redox mediators. However, the LRM‐based ECPB cannot free water electrolysis from dependency on membrane.^[^ ^27^ ^]^

Currently, the commercial low‐temperature OWS consists of alkaline water electrolysis (AWE) and proton‐exchange membrane water electrolysis (PEMWE). The AWE is of a mature technology and cost‐effective in instrumentation, but its major drawbacks are low theoretical efficiency and hard to apply for intermittent power sources. PEMWE compensates for the deficiency of AWE but suffers from a corrosive acidic electrolyte, which relies on precious metal catalysts for highly‐efficient and steady hydrogen production.^[^ ^28^, ^29^ ^]^ Lately, anion exchange membrane water electrolysis (AEMWE) is more appealing due to its advantages of adapting earth‐abundant and nonprecious metal electrocatalysts to promote HER in an alkaline medium and a high theoretical efficiency. However, high‐cost of AEM, poor ionic conductivity, sluggish mass transfer, and poor lifetime limit the practical applications of AEMWE at the moment.^[^ ^30^ ^]^

*iii) Solid‐state redox mediators (SRMs)‐based DWE*: In order to overcome the restriction of membrane toward OWS, the solid‐state redox mediators (SRMs) are then proposed to decouple the H_2_ and O_2_ without the use of membrane. Xia et al.^[^ ^31^ ^]^ firstly introduce the nickel hydroxide (Ni(OH)_2_/NiOOH) as SRMs to realize the separation of H_2_ and O_2_. In this architecture, the H_2_ production at the cathode accompanied by the oxidation of Ni(OH)_2_ to NiOOH at the anode, The following OER process involved reduction of NiOOH to Ni(OH)_2_. Following this conception, more SRMs such as MnO_2_/MnOOH,^[^ ^32^ ^]^ NaTi_2_(PO_4_)_3_,^[^ ^33^ ^]^ CuFe‐TBA,^[^ ^34^ ^]^ PANI,^[^ ^35^ ^]^ etc., with the redox potential windows located between HER and OER, are used for decoupled hydrogen production. Yang et al.^[^ ^34^ ^]^ report a CuFe Turnbull's blue analog (CuFe‐TBA) as SRM to decouple water electrolysis. Benefiting from the fast proton conduction in this structure, the decoupled system indicate high‐rate performance of 42.7 mAh g^−1^ at 120 A g^−1^ without the high‐cost membrane and can produce high‐purity hydrogen from renewable energies (Figure 2d). Zhou et al.^[^ ^36^ ^]^ introduce Mo foil oxidation reaction to water electrolysis system and achieve a low cell voltage of 0.598 V to reach the current density of 50 mA cm^−2^ and realize long‐term stability of 150 h at a constant voltage of 1.2 V. However, these approaches can hardly realize continuous working, require batch operation to regenerate the auxiliary SRMs electrodes. In addition, the capacity and rate limitations of SRMs have to be considered.^[^ ^37^ ^]^ Recently, Rothschild et al.^[^ ^38^ ^]^ propose the concept that the continuous operation in a membraneless decoupled system using NaBr as LRMs in water (Figure 2e). Bromide is electro‐oxidized to bromate accompanied by hydrogen production in one cell, bromate is chemically reduced to bromide and evolves oxygen in another cell. The whole process show high faradaic and electrolytic efficiency. However, it should be noted that although the membrane can be replaced by SRMs or LRMs, avoiding the gas crossover and risk of explosion, OER is still in existence and assists the electrocatalysis cycle. The only purpose of OER is to provide electrons to cathode for H_2_ producing and the O_2_ is merely by‐product. In addition, the OER is a sluggish process that requires majority of the energy input in OWS. In view of this, the review mainly focuses on the various strategies so that the OER can be efficiently circumvented.

**Figure 3:**
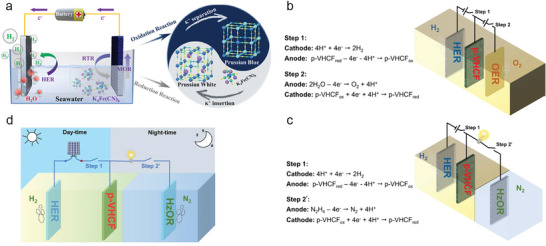
a) Schematic illustration of the ferrocyanide‐assisted prussian white/prussian blue redox‐coupled direct seawater electrolysis system for hydrogen production. Reproduced with permission.^[^ ^39^ ^]^ Copyright 2023. American Chemistry Society. b) Schematic illustration of the vanadium hexacyanoferrate redox mediator decoupled hydrogen/oxygen production from acidic water splitting. c) Schematic illustration of the vanadium hexacyanoferrate redox mediator decoupled hydrogen production and hydrazine oxidation. d) Schematic illustration of the decoupled system driven by collar cell. Reproduced with permission.^[^ ^40^ ^]^ Copyright 2024. Springer Nature.

*iv) Improvement of DWE*: As mentioned above, although the decoupled water electrolysis system introducing LRM/SRM redox mediates can avoid expensive membrane and H_2_/O_2_ mixed explosion risk, but the OER still be participated in the coupled system. The sluggish OER can inevitably increase electricity input, and the value of O_2_ is very limited compared with H_2_. Inspired by concept of above‐mentioned coupled water electrolysis system, the replacement of thermodynamically favorable small molecules oxidation by OER in decoupled water electrolysis can address above situation. Wu et al.^[^ ^39^ ^]^ introduce [Fe(CN)_6_]^4−^ as a redox mediator to assist prussian white (PW)/prussian blue (PB) redox, which delivers an ultralow onset potential of 0.87 V vs RHE (**Figure** **3**a). The [Fe(CN)_6_]^4−^ can induce the instantaneous reduction of PB to form PW, thus realizing regeneration of anodic starting materials and redox cycle of PW/PB. This decoupled system can well avoid OER and obtain value‐added hydrogen and K_3_[Fe(CN)_6_] that can be used as fertilizer, corrosive agent, indicator, medicine, and many others. In addition, the assembled electrolyzer shows unprecedented performance with low electricity input (42% lower than OWS) for seawater splitting. Benefiting from the low reaction potential, the competing chlorine electrooxidation reaction (ClOR) can be suppressed, enabling high‐purity hydrogen production.

Recently, Chen et al.^[^ ^40^ ^]^ propose a decoupled water electrolysis system that contained preprotonated vanadium hexacyanoferrate (p‐VHCF) as a redox mediator to divide H_2_/O_2_ without membrane. More importantly, the HzOR is involved in this coupled system to realize electricity generation in night. The prototype of this coupled system composes of HER, OER, and p‐VHCF redox mediator (Figure 3b). Specifically, the HER couples with oxidation of p‐VHCF (p‐VHCF_ox_ → p‐VHCF_red_). On the contrary, the OER couples with oxidation of p‐VHCF (p‐VHCF_red_ → p‐VHCF_ox_). After the OER is replaced by HzOR, a new electrolysis architecture is constructed (Figure 3c). In view of the lower oxidation potential of HzOR than the reduction potential of p‐VHCF_ox_, they construct a p‐VHCF‐N_2_H_4_ liquid battery and enable the conception of flexible energy conversion and storage (Figure 3d). The solar energy‐derived water splitting achieves high‐rate hydrogen production at day‐time, while the p‐VHCF‐N_2_H_4_ liquid battery works at night‐time for electricity output. In addition to the design of above‐mentioned O_2_‐free DWE, the following sections elaborate on the coupled water electrolysis systems and electrocatalyst design based on the value‐added and thermodynamically favorable oxidation reactions, to replace OER.

## 2 Coupled Water Electrolysis Electrocatalysts and Systems Design

### 2.1 Rational in Electrocatalyst Design

Small molecule oxidation (SMO) coupled water electrolysis shows promising for low‐energy‐consumption H_2_ production and chemical upgrading or pollutant treatment. However, the activity and stability of SMO are far from satisfactory for industrial applications. The drawbacks include high overpotential compared with the thermodynamic potential of the target oxidation reactions, low current density, and poor durability. For example, the low thermodynamic potentials of hydrazine oxidation reaction (HzOR, –0.33 V vs RHE) and urea oxidation reaction (UOR, 0.37 V vs RHE) are appealing for low‐energy‐consumption H_2_ production. Many efforts are devoted to exploring highly efficient and stable electrocatalysts for promoting these two SMOs, but most of the reported overpotentials are still higher than 200 mV for HzOR and 800 mV for UOR. The current density is still lower than industrial level (>1 A cm^−2^) (Table 3). There is still much room for improvement in efficiency, selectivity, and stability of electrocatalysts for SMO. The reaction thermodynamics and kinetics should be well promoted by careful regulation of the electronic structure, optimization of the adsorption energies of intermediates, creation of multiple active sites, and tailoring of morphology and interfaces of heterostructures. Hence, in this section, the design strategies of electronic structure regulation and morphological engineering are summarized and discussed for the electrocatalyst engineering for target SMOs.

#### 2.1.1 Electronic Structural Regulation

Tailoring of electronic structure of active sites is a powerful method of optimizing binding energies of key reaction intermediates, so as to enhance intrinsic catalytic performance for a target SMO.^[^ ^41^ ^]^ In this section, the strategies for tuning the electronic structures of electrocatalysts including heteroatom doping, heterogeneous structure engineering, and defect engineering are summarized and discussed.

##### Heteroatom Doping

Heteroatom doping has been well verified as an effective method for regulating the electronic structures and physicochemical properties of electrocatalysts. Such a useful strategy can highly improve their electrochemical performances in SMO. Heteroatom doping enriches active sites in electrocatalysts due to the mismatch of atomic radii, electronic configurations, and electronegativities. Doping with heteroatom usually induces atomic re‐arrangement and regulates the local electronic structure of heteroatom, and thereby improves catalytic activity.^[^ ^42^, ^43^ ^]^ The strategy for heteroatom doping generally involves two categories: nonmetallic and metallic. For instance, Huang et al.^[^ ^44^ ^]^ immobilize atomic Ni into In_2_O_3_ (Ni–In_2_O_3_) by a hydrothermal and controlled annealing strategy to promote UOR. In their contribution, the heteroatom doping of Ni atoms leads to the synergistic effect between Ni and oxygen vacancies (Vo). The Vo formation induced electron delocalization of the Ni–N bonds, accommodated around other bonds. In this regard, N atoms in *CO(NH_2_)_2_ prefer to be adsorbed on two adjacent Ni atoms to promote the rate‐determining step (RDS) of *CO(NH_2_)_2_ → *CONHNH_2_. As a result, the Ni–In_2_O_3_ achieved a current density of 25 mA cm^−2^ with a low potential of 1.31 V versus RHE for UOR, much lower than that of the pristine In_2_O_3_ (1.70 V vs RHE) at the same current density. The Ni–In_2_O_3_ electrocatalysts can also reach higher current density of 200 mA cm^−2^ at a required a bias of 1.42 V versus RHE. The highly‐efficient UOR performance is attributed to the synergy of the unique Ni–Vo structure to reduce the energy barrier of UOR.

Cui et al.^[^ ^45^ ^]^ design a nickel–cobalt–chromium layered double hydroxide (NiCoCr‐LDH) supported on nickel foam by simple one‐pot hydrothermal reaction for UOR. The Cr‐doping can optimize the UOR potential to as low as 1.38 V versus RHE to reach 100 mA cm^−2^ compared with non‐Cr NiCo‐LDH (≈1.44 V vs RHE). The potential of NiCoCr‐LDH reduces by 250 mV compared with OER, indicating a lower energy input of UOR than OER on NiCoCr‐LDH electrocatalysts. XAS and in situ Raman reveal that Cr‐doping modified the local coordination structure and electronic state of active sites. DFT calculations show that the Cr doping not only enhances the adsorption of urea substrates, but also favors the formation of UOR intermediates via decreasing RDS of UOR. In addition, the unique electronic structures brought by Cr atoms facilitate the in situ formation of *γ*‐NiOOH via surface structure reconstruction. Such a phase transformation is driven by narrowing the hybridization between H‐1s orbitals and O‐2p_z_ orbitals. As a result, a two‐electrode UOR||HER system delivers a current density of 10 mA cm^−2^ only required a cell voltage of 1.427 V, reduced by 97 mV compared with the traditional OWS. The UOR||HER system also maintains for 45 h in alkaline urine.

Single atoms catalysts (SACs) are advantageous and attracted particularly attention because of atomic efficiency for catalysis. This is important for rare metals and precious catalysts to maximize the usage so that the mass activity can be improved dramatically.^[^ ^46^ ^]^ The early studies mainly focused on carbon‐based supports because the heavier single atoms can be easily detected via electron microscopy. This provides a fitting guidance for exploring other 2D supports such as metals, oxides, sulfides, and metallenes.^[^ ^47^, ^48^ ^]^ Doping single‐atom in metal‐based supports reveals the active role of the support, compared with passive carbon, due to the synergy between a dopant metal atom and the support atoms by electronic metal–support interactions. Luo et al.^[^ ^49^ ^]^ develop Ru single atoms doping in Cu lattice and in situ grown on copper foam (RuCu/CF) by surface oxidation, Ru ion exchange, and electroreduction approaches. The elaborate RuCu/CF catalysts show remarkable catalytic activity for furfural oxidation reaction (FOR) coupled with H_2_ production. Under a three‐electrode UOR system, the oxidative current density reaches 150 mA cm^−2^ at a low potential of 0.37 V versus RHE and delivers a high yield/conversion rate of ≈100% formate acid. The assembled HER||FOR electrolyzer shows an ultra‐low cell voltage of 0.43 V to attain current density of 100 mA cm^−2^. A novel self‐powered direct furfural fuel cell (DFFC) is also integrated, displaying a peak power density to 193 mW cm^−2^. The superior performance is attributed to the single Ru atom doping that regulates the electronic structure of RuCu to promote water dissociation (RDS), and the adsorption of ΔG_H*_ on RuCo can be also optimized to near‐zero for promoting alkaline HER. Wang et al.^[^ ^50^ ^]^ introduce a high Ru loading (5.41 wt%) on 2D MOFs (NiRu‐ABDC). The highly‐efficient HzOR activity is achieved at a low potential of –83 mV to reach 10 mA cm^−2^. A two‐electrode cell assembling NiRu‐ABDC as a bifunctional catalyst exhibits cell voltages of 0.029 and 0.15 V at 10 and 100 mA cm^−2^, respectively, outperforming the state‐of‐the‐art Pt/C catalysts (*η*_10_ = 0.12 V, *η*_100_ = 0.66 V). DFT calculations reveal that the Ru doping induces up‐shift of the *d*‐bond center, accelerating adsorption of various intermediates (H_2_O*, OH*, and H* in alkaline HER) and the stepwise dehydrogenation of N_2_H_4_ for HzOR.

In addition to metallic atom doping, SMO performance has also been proved via nonmetallic doping. Zhang et al.^[^ ^51^ ^]^ design an N‐doped CoP supported on conductive cloth (CC@N‐CoP) as novel catalysts for sulfion oxidation reaction (SOR) coupling H_2_ production. Benefiting from the strong electronegativity of N, compared to P, the *d*‐bond center of CoP is decreased, which weakened the H* adsorption to a more thermoneutral state. As a result, an overpotential of 42 mV is obtained to drive ≈10 mA cm^−2^ for HER. The assembled HER||SOR mediated by the Fe^2+^/Fe^3+^ redox delivers 10 mA cm^−2^ at a cell voltage of only 0.89 V, along with a high sulfur production efficiency of 95.1%. This system is promising for low‐energy‐consumption H_2_ production and sulfur sources recycling.

##### Heterogenous Structure Engineering

Heterostructure electrocatalysts are hybrid materials with heterogeneous interfaces. This unique structure holds significant advantages from synergy between the constituents involved, such as promoted interfacial contact for efficient electron transfers, adjustable electronic structure and physical/chemical properties. Hence, delivering highly‐efficient electrochemical performance.^[^ ^52^, ^53^ ^]^ The hybrid materials may be classified into various categories, such as metal–carbon, inorganic‐compound, and multimetallic structures. For metal–carbon structures, the conductive carbon‐based materials are appealing substrates to support metallic nanoparticles due to the admirable intrinsic electrical conductivity to promote charge transfer processes across the carbon‐metal interface, and can retard agglomeration of nanoparticles.

Xie et al.^[^ ^54^ ^]^ construct a Ni/C hybrid nanosheet array with dual nanoislands including the part of pure Ni nanoparticles and the other part is core–shell Ni@C structure. The exposed Ni atoms in pure Ni nanoparticles and Ni/C structure are the active sites for promoting HzOR and HER. To reach a current density of 10 mA cm^−2^, the working potentials of –20 and –37 mV are obtained for HzOR and HER, respectively. The integrated HzOR||HER electrolyzer only requires 0.14 V to reach 50 mA cm^−2^. The well‐adjustable electronic structure of neighboring carbon atoms can be precisely regulated by the anchored metal particles. DFT calculations show that the bifunctional properties of the dual nanoislands on Ni/C are attributed to the moderation of H* adsorption on the carbon sites for HER, and of the dehydrogenation kinetics on exposed Ni sites for HzOR. Qu et al.^[^ ^55^ ^]^ propose Ru nanoparticles supported on mesoporous N‐doped carbon (Ru/MPNC) for bifunctional HER and HzOR performance. The highly exposed Ru particles are well confined in carbon network via Ru–N bonds. As a consequence, only –39 and 72 mV are required respectively at 10 and 100 mA cm^−2^ on Ru/MPNC for HzOR, which is much lower than the commercial Pt/C (137 and 301 mV) at the same current densities. A two‐electrode HER||HzOR cell is constructed. To attain10 and 50 mA cm^−2^, the system only requires 41 and 149 mV on Ru/MPNC, respectively. In addition, benefitting from the confined effect of carbon matrix to prevent Ru particles from aggregation, the Ru/MPNC exhibits long‐term stability to maintain 10 h at a high current density of 100 mA cm^−2^. DFT calculations show that the incorporation of Ru particles with N‐doped carbon induces an “up” shift of the *d*‐bond center. The created interfaces between the carbon substrate and the Ru particles contribute to the optimization of the electronic structure of Ru to lower the RDS (N_2_* to N_2_) of HzOR.

For inorganic‐compound structures, the typical cases are metal oxide‐based. Yin et al.^[^ ^56^ ^]^ introduce NiCo_2_ nanowire‐supported MoO_2_ to promote 5‐hydroxymethylfurfural oxidation reaction (HMFOR) assisted H_2_ production. It is identified that the HMF dehydrogenation kinetics is promoted by MoO_2_ due to accelerated electron/proton transfer in HMFOR, and further regulated adsorption behavior of HMFOR intermediates. The evidence is noted from the difference in electron densities and the total density of states, due to the increased states around the Fermi level from the construction of NiCo_2_@MoO_2_ heterostructures. This result indicates a higher conductivity of NiCo_2_@MoO_2_. Partial density of states shows that the 3d orbitals of Ni and Co are close to *E*_f_, suggesting that the Ni/Co sites are responsible for accelerating the electron transfer and lowering the energy barrier of HMFOR dehydrogenation (weaken the C−H/O−H bond). While a broad shape of Mo 4d orbital is conductive to stabilize Ni/Co valence and promote the adsorption of HMF and their intermediates. As a result, a low potential is obtained of 1.20 V versus RHE to reach 10 mA cm^−2^ with a high selectivity to furandicarboxylic acid (FDCA, 99.2%). Such an activity is much superior to those of the individual component NiCo_2_ (1.24 V vs RHE) and MoO_2_ (≈1.35 V vs RHE). The integrated two‐electrode HMFOR||HER system delivered 10 mA cm^−2^ with an ultralow cell voltage of 1.25 V. This system can maintain five consecutive conversion tests with a good stability.

The multimetallic composition structures generally include phosphide‐, chalcogenide‐, and nitride‐based heterostructures to reduce dependence on noble metals. Yang et al.^[^ ^57^ ^]^ establish an bifunctional Mn@Ni_3_N‐Co_3_N supported on nickel foam for HzOR and HER. Structural characterizations demonstrate the formation of tight interface between Ni_3_N and Co_3_N with highly matched lattices. For example, the work functions (*W*_f_) of Ni_3_N and Co_3_N are calculated to be 4.14 and 4.44 eV by ultraviolet photoelectron spectroscopy (UPS) spectroscopy, respectively. The contact between Ni_3_N and Co_3_N induced high‐*E*_f_ free electrons of Ni_3_N diffusing into the low *E*_f_ spontaneously, indicating the well lattice matching between Ni_3_N and Co_3_N and forming an unblocked internal conductive pathway to accelerate charge transfer. In addition, the Mn doping decreases the difference of *W*_f_ between Ni_3_N and Co_3_N, thus, proving the electron transfer from Ni_3_N to Co_3_N along the interfaces. The electron redistribution in Ni_3_N/Co_3_N heterostructure contributes to the promoted HzOR and HER activity. As a consequence, only 0.49 V cell voltage is required to reach industrial‐level current density (500 mA cm^−2^) on Mn@Ni_3_N‐Co_3_N/NF. This HzOR||HER coupled system saves 53.3% of electricity input for H_2_ production compared with OWS. Wu et al.^[^ ^58^ ^]^ propose an ultrafine Ni_2_P–Co_2_P heterostructures in a single nanosheet by topological transformation strategy. *Operando* Raman analyses reveal that the phosphide‐derived (oxy) hydroxide species (NiOOH–CoOOH) are the main active species to promote UOR. DFT calculations reveal the synergy between NiOOH and CoOOH, where the urea molecules prefer to adsorb on the Co sites, while Ni sites promotes CO_2_ product desorption. The UOR pathway is further uncovered: the Co sites reduce the energy barrier of UOR, demonstrating the synergistic effect between Co and Ni sites in NiOOH–CoOOH to catalyze UOR. As a result, the Ni_2_P–Co_2_P delivers a low potential of 1.27 and 1.32 V versus RHE at 10 and 100 mA cm^−2^, respectively. The Ni_2_P–Co_2_P outperforms the individual component of Ni_2_P (≈1.43 V vs RHE) and Co_2_P (≈1.37 V vs RHE) to reach 100 mA cm^−2^, indicating the well‐balanced electronic interactions in the Ni_2_P–Co_2_P heterostructure.

Wang et al.^[^ ^59^ ^]^ construct iron molybdenum sulfide (FeMo‐S) nanosheets as substrates to support ultrafine ruthenium (Ru) nanoclusters (FeMo‐S/Ru)) for sulfion oxidation reaction (SOR) coupled with H_2_ production. The integrated SOR||HER electrolyzer achieves a cell voltage of 0.57 V to reach 100 mA cm^−2^ and also exhibits long‐term stability of 838 h. In situ Raman together with DFT calculations uncover the SOR and HER mechanisms. The interfaces created by FeMo‐S/Ru heterostructures facilitate the water dissociation (RDS) in alkaline HER, while the Ru sites are active centers for SOR (S^2‐−^ → S). In addition, the strong interaction between FeMo‐S and the electron‐deficient Ru contributes largely to the long‐term stability of the electrolysis.

##### Defect Engineering

The incorporation of defects in electrocatalysts is considered to tailor the surface properties, largely modify the local electron configuration and create extra energy levels of density of states between conduction and valence bands, altering the intermediate species to an appropriate level.^[^ ^4^ ^]^ The strategy for defects engineering includes vacancies, strains, and lattice disorder, etc. Yang et al.^[^ ^60^ ^]^ develop an oxygen vacancies modified CuO nanorod array supported on copper foam (Vo‐rich CuO/CF) via fast calcination process in Ar for electrooxidation of benzylamine (BA) to benzonitrile (BN) assisted H_2_ production. In their control experiments, the Vo‐poor CuO/CF is prepared by direct annealing of the commercial CF in air at 250 °C and then subjected to BA oxidation in a three‐electrode system. A low potential of ≈0.5 V versus Ag/AgCl is attained on the Vo‐rich CuO/CF in 1.0 M KOH + 20 mM BA electrolyte, to reach 10 mA cm^−2^, which is about 100 mV lower than that of Vo‐poor CuO/CF. In situ Raman and FTIR together with DFT calculations indicate that the presence of Vo is in favor of adsorbing OH* species and BA substrate molecules, promoting the kinetics of BA dehydrogenation. The fundamental principle is attributed to that the Vo led to electron transition from valence band to the conduction band and the *d*‐band center of Vo‐CuO is shifted toward the Fermi level. This electron configuration effect is conducive to the optimal adsorption of OH* and reaction intermediates to accelerate the BA oxidation.

The regulation between reactants and excessive oxygen vacancies is still challenging. Zou et al.^[^ ^61^ ^]^ construct S element‐filed Ov with disordered structures in a layered double hydroxide (S‐Ov‐LDH) for HMFOR. The oxidative potential of HMFOR is significantly reduced and the current density is elevated. Compared with S‐free Ov‐LDH, the S‐Ov‐LDH delivers current densities of 10 and 50 mA cm^−2^ at relatively low potential of 1.26 and 1.39 V versus RHE in 1.0 M KOH + 50 mM HMF for HMFOR. Structural characterizations and DFT calculations elucidate the superior HMFOR on S‐Ov‐LDH as due to the formation of metal–S bonds induced by Ov defect‐filling to regulate the electronic structures around the Fermi level, weakening the over‐strong adsorption of OH* and HMF, leading to the formation of Co^3+^ active centers for efficient HMFOR. In addition, the introduction of lattice strains can cooperatively contribute to the SMO process. Wang et al.^[^ ^62^ ^]^ construct n‐type semiconductor NiO/Ru heterostructures with ≈0.5–2% lattice tensile strains that flatten the gamma band around Fermi level, hence, increasing the level of charge concentration. Therefore, the electron conductivity is improved significantly for SMO catalysis. As a result, the Ru_13_ top sites are the active centers in the NiO/Ru heterostructures for HER and HzOR by reducing the RDS of *NH_2_NH_2_ to *NH_2_NH for HzOR and optimizing ∆*G*_H*_ for HER. As a result, the NiO/Ru drives the current densities to10 and 100 mA cm^−2^, respectively, at an ultralow cell voltage of 0.021 and 0.22 V for HzOR||HER. As a sharp contrast, the traditional OWS delivers 10 mA cm^−2^ at a much higher cell voltage of 1.58 V. The design of semiconductor catalysts with strains provided a novel insight for SMO process.

#### 2.1.2 Morphological Engineering

In addition to the intrinsic activity regulation via electronic structure engineering, the apparent activity is promoted by increasing active sites number via morphological engineering. Accordingly, the electrocatalysts are classified as zero‐dimensional (0D, nanoparticles), one‐dimensional (1D, nanowire, nanocube, etc.), two‐dimensional (2D, nanosheet), and three‐dimensional (3D, 1D/2D/3D composite structures). The specific dimensions would exhibit high surface energy with relatively large surface area and mass transfer, which dominated dramatically under an industrial‐scale current density. 0D electrocatalysts possess wide energy gaps and surface energy, enabling discontinuous HOMO and LUMO levels for promoted SMOs catalysis.

Choi et al.^[^ ^63^ ^]^ report a spherical‐shaped AuPt nanoalloys by laser irradiation as bifunctional electrocatalysts for HzOR‐assisted HER. The surface area of AuPt nanoalloys is calculated to be ≈293.3 m^2^ g^−1^. The large specific surface area of AuPt nanoalloys endow them better adsorption of reactant and intermediates. The HzOR potential is 502 mV to reach 10 mA cm^−2^. The assembled HzOR||HER two‐electrode system using AuPt alloys as both anode and cathode electrodes, delivers a cell voltage of 0.172 V at 10 mA cm^−2^, outperforming traditional OWS of 1.773 V at the same current density. The polyol template method can assemble 0D nanomaterials to 2D structure with wavy surface for exposing more active sites. Duan et al.^[^ ^64^ ^]^ propose a RhRu_0.5_ wavy nanowires that is composed of 0D nanoparticles as bifunctional electrocatalysts for HzOR and HER. Thanks to the large electrochemically active surface area and good charge transport along the 1D nanowires, the enriched active sites are realized to promote mass activity of RhRu_0.5_ (60.4 ± 6.2 A mg^−1^ at 0.20 V vs RHE for HzOR), reaching about one order of magnitude larger than commercial Pt/C catalysts. The HzOR‐assisted OWS system reached high current density of 100 mA cm^−2^ requires an ultralow cell voltage of 54 mV. This RhRu_0.5_ nanowire also maintained a stable operation for 80 h at 100 mA cm^−2^. 2D metal nanomaterials owns ordered atom arrays to enable necessary conductivity and the created porous structure leads to large surface area to contact with electrolyte, thereby, exposing more active centers.

Considering the main challenge of activating inert in‐plane atoms of 2D materials, Dai et al.^[^ ^65^ ^]^ design mesoporous PtPb nanosheets by a one‐pot hydrothermal method. The ethylenediamine and Pd are the key component to create 2D nanosheets structure, while ethylenediamine tended to chelate with Pt^2+^ and Pb^2+^ to form nanosheets and thereafter Pt is reduced in the nanosheet to create porous structure. The unique mesoporous PtPb nanosheets showed superior methanol oxidation with 5.66 times current density greater than that of commercial Pt/C. 3D structures mainly composed of multiple 0/1/2D nanomaterials. One of the main strategies is to construct self‐supporting and superhydrophilic/superaerophobic hierarchical structures, because the contact behavior is crucial for exposing activate electrocatalysts by improving contact area between catalysts and electrolyte. In addition, the capacity of gas bubble detachment is related to the mass transfer kinetic under high current density. The superhydrophilic/superaerophobic surface can be created using various 3D morphologies, such as the assemble of 2D nanosheets, nanowires, and nanotubes, etc., to form 3D cross‐linking or nanoarray hierarchical structures.^[^ ^66^, ^67^ ^]^

Liu et al.^[^ ^68^ ^]^ construct amorphous RuMo alloy nanoclusters integrated with amorphous NiMoO_4_ skeletons and supported on nickel foam (*a*‐RuMo/NiMoO_4_/NF) for HzOR‐assisted OWS. The as‐prepared *a*‐RuMo/NiMoO_4_/NF shows 3D nanorod array morphology with hollow structure in these nanorods. Such a novel 3D hollow nanoarray structure with amorphous feature is attributed to the incorporation of Ru^3+^ induces migration of Mo in NiMoO_4_ during annealing process, interfered atomic sequence of NiMoO_4_ and led to the amorphous feature of NiMoO_4_. In addition, the external Mo atoms interfered atomic sequence of Ru to form amorphous RuMo alloy. The *a*‐RuMo/NiMoO_4_/NF also shows superhydrophilic nature (≈0° of contact angle) compared to commercial nickel foam (74.9°). As a result, the novel 3D hollow structure and amorphous RuMo/NiMoO_4_ heterostructures deliver as low as –91 and 276 mV to reach 10 and 500 mA cm^−2^, respectively. The assembled HzOR||HER electrolyzer using RuMo/NiMoO_4_/NF as bifunctional electrocatalysts exhibit low cell voltages of 7 and 420 mV to reach 10 and 500 mA cm^−2^, and maintain 100 h to reach high current density of 500 mA cm^−2^, demonstrating the potential application of the *a*‐RuMo/NiMoO_4_/NF for HzOR‐assisted OWS for low‐energy‐consumption H_2_ production.

### 2.2 Coupled Water Electrolysis System Design

Following the aforementioned DWE drawbacks in Section 1 brought by OER, Sun et al.^[^ ^14^, ^69^, ^70^ ^]^ propose concept of coupled OWS, in which the OER is replaced by thermodynamically favorable small molecules oxidation and coupled with HER. Such a novel coupled OWS realizes several advantages: i) The energy conversion efficiency can be improved due to the higher delivered current density under lower cell voltages. ii) The value‐added reaction process rather than O_2_ and ROS can be obtained, improving the return of energy investment and increase of life for electrolyzers. iii) H_2_/O_2_ explosion risk can be circumvented due to no O_2_ will be produced. The substitute small molecules should satisfy the following requirements: i) The starting substances should be soluble in aqueous media. ii) The final product should be more valuable than raw materials. iii) The thermodynamic potential should lower than that of OER.

#### 2.2.1 Value‐Added Chemicals Upgradation Oxidation Reaction

##### 5‐Hydroxymethylfurfural (HMF) Oxidation

**Figure 4:**
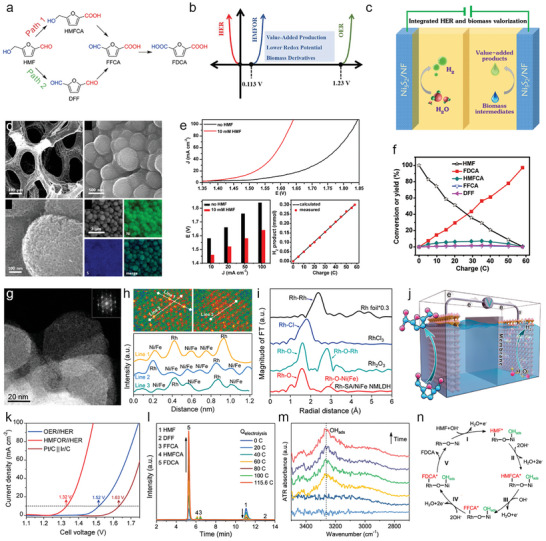
a) A general two pathways for the synthesis of FDCA products from HMF stating substances. b) Thermodynamic potentials of HMFOR, HER, and OER. Reproduced with permission.^[^ ^75^ ^]^ Copyright 2022. Wiley‐VCH. c) Schematic illustration for HMFOR‐based coupled OWS. d) SEM of hierarchically porous Ni_3_S_2_/Ni foam and elemental mappings. e) LSV curves of coupled system with or without the addition of 10 mM HMF, the corresponding cell voltages at the specific current densities (bottom left), and the Faradic efficiency for H_2_ production (bottom right). f) Conversion of yields versus charge plots of HMF and the products. Reproduced with permission.^[^ ^70^ ^]^ Copyright 2016. American Chemistry Society. g) HAADF‐STEM and corresponding FFT images of Rh‐SA/NiFe NMLDH. h) Processed HAADF‐STEM images. i) Fourier‐transformed EXAFS curves of Rh *K*‐edge. j) Schematic illustration of HMFOR‐assisted H_2_ production system. k) LSV curves of the coupled OWS and conventional OWS. l) HPLC of anodic HMFOR obtained at various electrolysis charges. m) *Quasi‐operando* ATR‐FTIR spectra. n) Proposed HMFOR mechanism. Reproduced with permission.^[^ ^76^ ^]^ Copyright 2023. American Chemistry Society.

Based on the above criterions, lignocellulosic biomass has received concentrated attention in recent years because it is the bio‐renewable carbon‐neutral resource and the most abundant in spacious geographical distribution.^[^ ^71^ ^]^ Among them, the lignocellulose biomass or fructose‐derived 5‐hydroxymethylfurfural (HMF) is a kind of acid‐catalyzed dehydration product of C6 carbohydrates for a diversified applications, such as hydrogenation, catalytic oxidation, and condensation to synthesize fuels and high energy density chemicals.^[^ ^72^ ^]^ The oxidative products of HMF are various of 5‐hydroxymethyl‐2‐furancarboxylic acid (HMFCA), 2,5‐diformyl furan (DFF), 5‐formyl‐2‐furancarboxylic acid (FFCA), and 2,5‐furandicarboxylic acid (FDCA) (**Figure** **4**a). Each of them is used in diversified applications for polyester synthesis, fuel, chemical intermediates, antifungal agents, drugs, etc.^[^ ^73^, ^74^, ^75^ ^]^ The selective HMFOR to various products of HMFCA, DFF, FFCA, and FDCA is controllable. Generally, HMFCA is the main product at low potential (≤0.4 V), while FDCA can be formed at a relatively high potential (≥1.0 V) under a strong alkaline medium (pH ≥ 13).^[^ ^75^ ^]^ The formation of DFF usually occurs in a mild neutral condition due to the low OH^−^ concentration that cannot hinder the adsorption of HMF molecules on the catalyst surface. It should be noted that the narrow oxidative potential range of FFCA and the competition with FDCA leads to a low selectivity of FFCA (≤60%). Catalyst selectivity is also crucial for selective HMFOR. The detailed information of catalysts design and reaction conditions for optimal yield, selectivity, and conversion rate of HMFOR are listed in **Table** **1**.

**Table 1:** Comparison of various oxidative products of DFF, HMFCA, FFCA, and FDCA.

Products	Catalysts	Electrolytes/ HMF concentration (mM)	*E* (V vs RHE)	Yield/Conv. or Sel. (%)	Ref.
FDCA	S‐Ov‐LDH	1.0 M KOH/50	1.26	98.4/99.1	[61]
FDCA	A‐Co‐Ni_2_P	1.0 M KOH/100	1.50	99.2/99.4	[95]
FDCA	Pd‐NiCo_2_O_4_	1.0 M KOH/50	1.50	–/99.2	[96]
FDCA	Pd/NiFe	1.0 M KOH/10	1.50	97.9/98.8	[97]
FDCA	Rh‐SA/NiFe NMLDH	1.0 M KOH/50	1.35	–/98.0	[76]
FDCA	Ru@Ni_x_Co_1‐x_(OH)_2_	1.0 M KOH/5	1.40	≈100/≈100	[88]
FDCA	pCoHA‐Ru	1 M KHCO_3_/100	1.45	92.1/–	[98]
FDCA	Co@NiMoO‐Ni/NF	1.0 M KOH/10	1.40	98.6/–	[86]
FDCA	NiCo_2_@MoO_2_/NF	1.0 M KOH/10	1.30	99.2/99.0	[56]
FDCA	Co_0.4_NiS@NF	1.0 M KOH/10	1.45	–/100	[80]
FDCA	Mn_0.2_NiS/GF	1.0 M KOH/100	–	97.6/98.3	[72]
FDCA	Ni‐VN/NF	1.0 M KOH/10	1.40	99.0/99.3	[93]
FDCA	NiFe‐1	1.0 M KOH/50	1.47	92.0/≈90	[99]
FDCA	CoP‐CoOOH	1.0 M KOH/150	1.42	–/99.4	[100]
FDCA	Ni_3_S_2_‐MoS_2_/NF	1.0 M KOH/20	1.70	96–97%/–	[87]
FDCA	CoNiP‐NIE	1.0 M KOH/10	1.70	85.8/–	[94]
FDCA	Cu(OH)_2_/NiOOH	0.1 M KOH/50	1.90^a)^	97.7/–	[91]
DFF	Ru_1_‐NiO	1.0 M PBS/10	1.50	42.5/72.4	[101]
DFF	Co_8_Ce_2_O*_x_*	1.0 M PBS/50	1.50	≈18/90	[102]
DFF	MnO*_x_*	H_2_SO_4_ (pH = 1)/20	2.0	41.9/43.7	[103]
DFF	PtRu	0.1 M H_2_SO_4_/100	–	22.2/89.0	[104]
DFF	Pt/Fe_3_O_4_/rGO	0.1 M K_2_SO_4_/25	0.60^b)^	6.8/94.4	[105]
DFF	TEMPO	0.5 × 10^−3^ M LiClO_4_/0.25	1^c)^	78.0/100.0	[106]
DFF	4‐AcNH‐TEMPO	0.2 m KI, 0.5 m NaHCO_3_, 0.4 M Na_2_SO_4_/50	80^d)^	64.0/69.0	[107]
FFCA	Cu NPs	0.1 M KOH/10	1.23	–/67	
HMFCA	Au/C	0.1 M KOH/20	0.60	98.0/98.0	[108]
HMFCA	Au_14_	1.0 M KOH/5	0.82	23.6/56.9	[109]
HMFCA	Cu	1.0 M KOH/50	0.40	≈70.0/100	[110]
HMFCA	Ru_1_‐NiO	1.0 M KOH/50	1.30	≈5.0/74.0	[101]
HMFCA	CoO*_x_*	1.0 M KOH/5	1.60	–/48.0	[102]

^a)^ Cell voltage;

^b)^ Versus Ag/AgCl;

^c)^ Current (mA);

^d)^ Current Density (mA cm^−2^)

As one of the major oxidation productions, FDCA can be used as an important precursor for producing polyethylene furandicarboxylate (PEF) in plastics industry to replace petroleum‐derived polyethylene terephthalate.^[^ ^76^ ^]^ Traditionally, HMF oxidation mainly relied on thermal catalysis, which required high elevated temperature (100–200 °C) and pressure (10 bar O_2_) as well as noble metal‐based catalysts (Ru, Rh, Pt, Pd, etc.). In addition, organic oxidant (e.g., K_2_Cr_2_O_7_ and KMnO_4_) and solvent are involved in the synthesis process, which inevitably introduces by‐products and extra purification process, increasing the cost and energy consumption of the production. Such a strategy violates the concept of economic and green producing.^[^ ^77^, ^78^ ^]^

Among the sustainable and ecological‐friendly oxidation strategies, electrooxidation is a promising pathway for the efficient conversion of HMF to aforementioned four compounds due to the green and energy‐effective synthetic condition.^[^ ^79^ ^]^ More gratifyingly, HMF electrooxidation reaction (HMFOR) takes priority in thermodynamic potential of only 0.113 V versus RHE than that of OER (1.23 V vs RHE), revealing promising alternative oxidation reaction for low‐energy‐consumption hydrogen production coupled OWS (Figure 4b).^[^ ^80^, ^81^ ^]^ In this regard, Sun and co‐authors firstly introduce the HMFOR in the coupled OWS to produce value‐added FDCA products (Figure 4c).^[^ ^70^ ^]^ In their coupled systems, hierarchically porous Ni_3_S_2_/Ni foam are used as bifunctional self‐supporting catalysts for anode and cathode (Figure 4d). Such a unique porous nanoarchitecture is able to increase the number of catalytically active sites and benefit mass transport for promoting electrochemical activity.^[^ ^82^, ^83^, ^84^, ^85^ ^]^ After integrating HER and HMFOR, the coupled OWS shows ultralow cell voltage of 1.64 V to achieve 100 mA cm^−2^ with ≈98% Faradic efficiency, saving ≈200 mV relative to conventional OWS, implying a higher energy conversion efficiency and achieving low‐energy‐consumption H_2_ production (Figure 4e). The anodic products are analyzed by high‐performance liquid chromatography (HPLC), the result showed 100% Faradic efficiency and 98% HMF conversion to FDCA production (Figure 4f). This new coupled water electrolysis strategy realizes the green electrosynthesis of FDCA value‐added chemicals and low‐energy‐consumption H_2_ production. Since then, a booming development of HMFOR‐based H_2_ production systems is witnessed regarding to the high‐performance catalysts design (NiMoO‐Ni,^[^ ^86^ ^]^ RuCu‐CF,^[^ ^49^ ^]^ NiCo_2_@MoO_2_/NF,^[^ ^56^ ^]^ Rh‐SA/NiFe NMLDH,^[^ ^76^ ^]^ Ni_3_S_2_‐MoS_2_,^[^ ^87^ ^]^ Ru@Ni*_x_* Co_1−_ *_x_* (OH)_2_,^[^ ^88^ ^]^ etc.) and in‐depth mechanism analysis.

Regarding noble metals, recently, Guo et al.^[^ ^76^ ^]^ report rhodium single atoms (Rh SAs) loaded on NiFe layered double hydroxides (Rh‐SA/NiFe NMLDH) as bifunctional catalysts for alkaline HER and HMFOR. The atomic‐level of Rh on NiFe NMLDH is confirmed by high‐angle annular dark‐field scanning transmission electron microscopy (HAADF‐STEM) images and Fourier‐transformed extended X‐ray absorption fine structure (EXAFS). The bright contrast spots can be identified as Rh SAs, the corresponding atomic line profiles along the dash‐line 1, 2, and 3 validated the atomic dispersed of Rh SAs (Figure 4g,h). The FT‐EXAFS of Rh *K*‐edge indicates the coordination environment of Rh and Ni (Fe) atoms bridging with O atoms without the detection of metallic or oxidized Rh (Figure 4i). The integrated coupled OWS system indicates ultralow cell voltage of 1.32 V to achieve 10 mA cm^−2^, which is much lower than those of conventional OWS (1.52 V) and commercial Pt/C||Ir/C catalysts (1.63 V) (Figure 4j,k), indicating the low‐energy‐consumption H_2_ production when the HMFOR is involved in OWS. The content of HMF decreases with the increase of charge, and the level of FDCA increased, suggesting the successful transformation of HMF to FDCA product (Figure 4l). The HMFOR mechanism is then investigated by *quasi‐operando* ART‐FTIR (Figure 4m). The OH_ads_ is detected with negligible structural changes of catalysts, indicating the OH_ads_‐participated oxidation route rather than “electrochemical–chemical” mechanism dominates the HMFOR. Together with the *operando* X‐ray absorption spectroscopic analysis, they propose the HMFOR mechanism that the HMF* and OH_ads_ are adsorbed on Rh SAs and Ni sites, respectively, with the further oxidation to produce FDCA products (Figure 4n).

Compared with noble‐metal based catalysts for HMFOR‐based coupled water electrolysis system, more attention have been focused on the non‐noble catalysts such as metals,^[^ ^89^ ^]^ silicide,^[^ ^90^ ^]^ hydroxides,^[^ ^81^, ^91^, ^92^ ^]^ sulfides,^[^ ^72^, ^80^ ^]^ nitrides,^[^ ^93^ ^]^ and phosphides,^[^ ^94^ ^]^ etc., due to its earth‐abundant and low‐cost natures. Lee et al.^[^ ^91^ ^]^ prepare Cu(OH)_2_/NiOOH composite catalysts for collaborative electrooxidation of HMF. In their study, the Cu(OH)_2_ reveals superior reactivity for the transformation of aldehyde to carboxylic acid, while NiOOH shows better reactivity for the alcohol oxidation to form aldehyde. The Cu(OH)_2_/NiOOH mixed electrodes show higher activity of HMFOR than those of individual Cu(OH)_2_ and NiOOH, revealing the synergistic effect of the collaborative catalysts. As a result, high yield of FDCA (98.3%) is realized at the applied potential of 1.4 V versus RHE. However, considering the small thermodynamic potential of HMFOR (close to 0 V vs RHE), most of the reported systems are operated at still higher cell voltage (>1.23 V) due to inherent restrictions. In view of this, Wang et al.^[^ ^89^ ^]^ propose furfural electrooxidation on Cu nanowires for low‐potential H_2_ production*** at ≈0 V versus RHE. Interestingly, the low‐potential furfural electrooxidation coupled with oxygen reduction reaction (ORR) can produce furoic acid and hydrogen simultaneously, and accompany by electricity generation. The calculated electricity output is ≈2 kWh m^−3^ of H_2_ produced at the current density of 50 mA cm^−2^, providing a transformative technology for biomass upgrading and H_2_ production from energy input to electricity output.

##### Alcohol Oxidation

In addition to the investigation of HMFOR‐assisted OWS to obtain value‐added FDCA products and pure H_2_ with a low energy consumption, other reactions such as alcohol oxidation has evoked intensive interest on account of the high value‐added oxidation products (e.g., carboxylic acids) and lower thermodynamic potentials (e.g., methanol (0.103 V), ethanol (0.057 V), and glycerol (0.003 V) vs RHE) (Figure 1b).^[^ ^111^, ^112^, ^113^ ^]^ However, the challenge lies in multisteps electron transfer leads to different reaction selectively with the formation of various products. Therefore, understanding the mechanism of alcohol oxidation and increasing the selectivity of the target products is vital for the development of producing value‐added chemicals and pure H_2_ from alcohol‐assisted water electrolysis. Methanol ($350 ton^−1^) has the simplest structure among the alcohols and has been long used as a starting materials to synthesize formate ($1300 ton^−1^) with higher value, which owns wide applications for rubber and pharmaceutical industries.^[^ ^114^, ^115^ ^]^ Nevertheless, the traditional synthetic methods to synthesize formate involve hazardous conditions including higher temperature (363 K) and strongly acidic solutions, which might decrease the yields of formic acid due to the deep oxidation.^[^ ^116^ ^]^ Impressively, the electrochemical methanol oxidation reaction (MOR) holds great potential for green and sustainable pathway for producing formate. Together with its lower thermodynamic potential than that of OER, the MOR can be an ideal alternative to the OER in coupled water electrolysis. In this context, the concurrent production of high‐valued formate and pure H_2_ can be realized over noble and non‐noble transition metal electrocatalysts.

Yang et al.^[^ ^117^ ^]^ report a Co‐doped Rh nanoparticles with a size of ≈1.94 nm as bifunctional catalysts for alkaline HER and MOR. The Co‐doping can accelerate Volmer step of water dissociation and Heyrovsky step for H_2_ evolution. In addition, the energy barrier of MOR (*CO + *OH → *COOH) is decreased to 0.69 eV on the Rh_112_Co_4_ surface. As a result, the two‐electrode cell of MOR‐assisted OWS indicates a cell voltage of 1.545 V at the current density of 10 mA cm^−2^, outperforming the commercial Pt/C||IrO_2_ (1.658 V), revealing a promising electrocatalysts for MOR and HER. In addition to the Rh‐based composites, other noble metal‐based electrocatalysts such as Pt_1.8_Pd_0.2_CuGa/C,^[^ ^118^ ^]^ intermetallic NiIr‐MOF,^[^ ^119^ ^]^ PdSn‐Mxene,^[^ ^120^ ^]^ PtCo@NC,^[^ ^121^ ^]^ Pd_7_IrB_x_/NG,^[^ ^122^ ^]^ PtCuFe alloy,^[^ ^123^ ^]^ m‐PtRh NSs, etc.,^[^ ^124^ ^]^ have proved a highly‐efficient removal of CO intermediates and promoted methanol dehydrogenation step. As another a class of catalysts, non‐noble metal‐based catalysts such as NiMn/Fe‐LDH,^[^ ^111^ ^]^ h‐NiSe/CNTs,^[^ ^125^ ^]^ Cu_2_O‐Cu@Ni_2_P/NF,^[^ ^126^ ^]^ require low‐energy‐consumption to drive high current density for MOF‐assisted water electrolysis system. Ni‐based catalysts have been widely used for MOR and many efforts have been devoted to investigate this nucleophile oxidation reactions.

**Figure 5:**
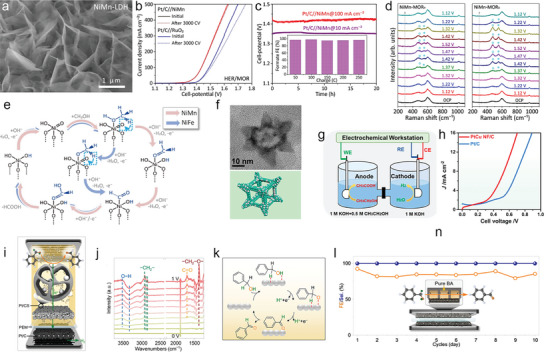
a) SEM image of NiMn‐LDH. b) LSV curves of two‐electrode electrolyze of HER|MOR on Pt/C||NiMn and Pt/C||RuO_2_ and the stability test after 3000 CVs. c) Chronopotentiometry tests of Pt/C||NiMn at 10 and 100 mA cm^−2^. d) *Operando* Raman spectra of NiMn‐LDH at various potential for MOR with H and D denotes solutions. e) The proposed bifunctional mechanism for MOR. Reproduced with permission.^[^ ^111^ ^]^ Copyright 2023. Springer Nature. f) TEM image and schematic model of PtCu nanoframe. g) Schematic of the two‐electrode EOR‐assisted HER system. h) LSV curves of the two‐electrode cell. Reproduced with permission.^[^ ^135^ ^]^ Copyright 2022. American Chemistry Society. i) Schematic illustration of all‐solid proton generator‐transfer cell for benzyl alcohol oxidation and H_2_ production. j) In situ ATR‐SEIRAS spectra for benzyl alcohol oxidation over cell voltage ranges from 0 to 1 V. k) Proposed mechanism for benzyl alcohol oxidation. l) Stability of the device at 1.2 V. Reproduced with permission.^[^ ^136^ ^]^ Copyright 2024. American Chemistry Society.

Recently, Feng et al.^[^ ^111^ ^]^ employ NiMn‐LDHs as the model catalysts for MOR (**Figure** **5**a). After integrating the MOR and HER using NiMn‐LDHs and commercial Pt/C as anodic and cathodic catalysts, respectively, the low cell voltages of 1.33/1.43 V are obtained to achieve 10/100 mA cm^−2^, and showed superior stability after 3000 CV tests (Figure 5b). The long‐term stability is further confirmed by chronopotentiometry (CP) tests for 20 h at 10 and 100 mA cm^−2^ (Figure 5c). The calculated Faradic efficiency of MOR to formate is ≈100% during the continuous operating. Based on the *operando* Raman tests (Figure 5d), the MOR mechanism on NiMn‐LDH involves two concomitant processes of reversible redox transformation between Ni^II^‐(OH)_2_ and Ni^III^OOH, and then the MOR process. Together with the DFT calculations, a cyclic pathway of MOR is proposed, where the initial oxidative production of Ni^III^OOH provides combined active sites for a series of methanol dehydrogenations (Figure 5e). It is noted that the transition metal‐based hydroxides provide insufficient conductivity and activity toward HER, which is compelled to design different catalysts for HER and MOR. The development of bifunctional catalysts for concurrent of HER and MOR such as Mo‐Co_4_N^[^ ^127^ ^]^ and Ni_3_S_2_/CNTs, etc.,^[^ ^128^ ^]^ are reported for the highly‐efficient MOR‐assisted OWS coupled water electrolysis.

As another important biomass‐related chemicals, ethanol is the most popular C2 liquid alcohol with high energy density (21 MJ L^−1^), low toxicity, and low cost, can be derived from the hydration of ethylene or biomass fermentation.^[^ ^113^, ^129^ ^]^ From the view of producing value‐added chemicals via ethanol refinery, the C2 pathway of ethanol oxidation reaction (EOR) to produce acetate, acetaldehyde, and ethyl acetate, etc., is desirable. Combined with thermodynamic favorable feature of EOR than OER, the electrochemical EOR‐assisted OWS shows great potential for low‐energy‐consumption H_2_ production and value‐added conversion of ethanol. In addition, the high pressure/temperature and hazardous gas emissions processes during traditional method of EOR can be avoid.^[^ ^130^ ^]^ Shen et. al.^[^ ^131^ ^]^ report alcohol‐water electrolysis process (e.g., ethanol) that is selectively transformed into value‐added carboxylic compounds, simultaneously, pure H_2_ is obtained at one‐third of the energy input required by a traditional OWS on Pt||Pd–(Ni–Zn)/C electrodes. After that, many highly‐efficient noble or non‐noble electrocatalysts are employed in EOR‐assisted OWS, such as noble metal aerogels (Au, Pd, Ru, Ag, Rh, Pt alloys),^[^ ^132^ ^]^ Pd_2_Ga/C,^[^ ^133^ ^]^ NiOOH‐CuO,^[^ ^129^ ^]^ mesoporous PtPb nanosheets,^[^ ^65^ ^]^ CoSe_2_‐Ni,^[^ ^134^ ^]^ etc.

Recently, Liu et al.^[^ ^135^ ^]^ introduce a three‐dimensional PtCu nanoframe (NF) prepared by dealloying strategy for bifunctional EOR and HER. The TEM image of PtCu NF corresponds well to the proposed model (Figure 5f). The coupled HER/EOR on PtCu NF catalysts show ultralow cell voltage of 0.58 V at 10 mA cm^−2^, outperforming the commercial Pt/C (0.75 V) under the same condition (Figure 5g,h), which are all lower than those of traditional OWS (1.88 and 1.92 V, respectively), revealing the highly‐efficient PtCu NF catalysts for energy‐saving EOR‐coupled HER system. Such a superior activity of HER and EOR are attributed to the interaction between Cu and Pt, novel high‐index facets feature, and 3D open structure of PtCu NF. Later, in the PtPb nanosheets system is reported by Dai et al., the EOR mechanism is revealed by DFT calculations that the immobilization of Pb promoted the interaction between oxygen atoms and surface of catalysts, which decreases the energy barrier of rate‐determining step (RDS) of EOR compared with pristine Pt electrode.^[^ ^65^ ^]^

Glycerol as low‐cost by‐product in biodiesel production industry has received particular attention and been considered as an ideal renewable material for C1–C3 value‐added intermediates from selective glycerol oxidation (GOR) such as dihydroxyacetone, tartronic acid, glycolic acid, etc.^[^ ^137^ ^]^ In addition, considering the lower theoretical oxidation potential than OER, the GOR is a promising alternative to OER to achieve energy‐saving H_2_ production. However, the activity and selectivity are depended on electrocatalysts which is easily poisoned during GOR. Therefore, the design of highly‐efficient electrocatalysts is crucial for GOR. Recently, Wen et al.^[^ ^112^ ^]^ design high entropy CoNiCuMnMo alloy (HEA) as noble metal‐free transitional metal catalysts for GOR. The HEA electrode shows high activity for GOR with an ultralow potential of 1.25 V versus RHE to reach 10 mA cm^−2^. The high Faradic efficiency of 90% can be obtained to produce formate. Impressively, the assembled two‐electrode cell using HEA and commercial RuIr/Ti is as alkaline anode and acidic cathode, respectively, showing superior activity of low cell voltage (0.55 V at 10 mA cm^−2^) and long‐term stability (300 h at 50 mA cm^−2^).

The possible surface reconstruction of HEA is investigated by self‐developed machine learning, revealing that the Mo atoms coordinated by Mn, Ni, and Mo are active centers. The GOR mechanism is discussed by EC‐MS, operando Raman spectroscopy, and ATR‐IR spectroscopy. The results indicate that the glycerol molecules are firstly oxidized to carboxylic ion‐containing products in the potential range of 1.22−1.62 V versus RHE. The initially formed glyceraldehyde intermediates are further oxidized to glycolate and formate at the potential higher than 1.47 V versus RHE. Lately, Xie et al.^[^ ^138^ ^]^ develop ternary NiVRu‐LDH nanosheets on 3D nickel foam by one‐pot hydrothermal reaction as the bifunctional catalysts for GOR and HER. The integrated GOR‐assisted HER system using NiVRu‐LDH as both anode and cathode catalysts exhibit 1.35 V at 10 mA cm^−2^, which is much lower than that of traditional OWS (1.50 V at 10 mA cm^−2^). Such a coupled system also show long‐term stability of 120 h at 1 A cm^−2^. The in‐depth GOR mechanism is investigated by *operando* Raman measurements. The possible GOR mechanism involves three processes: i) electrooxidation of Ni^II^ to Ni^III^, ii) oxidation of glycerol by Ni^III^ and hydroxyl group, iii) reduction of Ni^III^ into Ni^II^. The DFT calculations of free energy for GOR intermediates indicate that the C–C bond cleavage is the RDS for both NiVRu‐LDH and NiV‐LDH, and the Ru doping can facilitate thermodynamic behavior of GOR. Except for the Ru‐based catalysts, other catalysts such as MnO_2_/CP,^[^ ^139^ ^]^ Au@NiS_x_,^[^ ^137^ ^]^ NiFe‐LDH,^[^ ^140^ ^]^ CNs@CoPt,^[^ ^141^ ^]^ (Fe, Ni, Mn, Co) oxides,^[^ ^142^ ^]^ MoO*_x_*/Pt,^[^ ^143^ ^]^ NC/Ni‐Mo‐N/NF,^[^ ^144^ ^]^ etc., are developed for highly‐efficient GOR‐assisted coupled water electrolysis. Moreover, the electrooxidation of cycloalkanol,^[^ ^145^ ^]^ ethylene glycol,^[^ ^146^, ^147^, ^148^, ^149^ ^]^ sterol,^[^ ^150^ ^]^ etc., are used to integrated with HER for the expansion of diversified applications.

The various oxidative products of alcohol oxidation reactions and two‐electrode performance coupled with H_2_ production are collected and listed in **Table** **2**. Despite the considerable progresses, alcohol oxidation‐assisted H_2_ production usually rely on active intermediates (*OH) from water oxidation, which may lead to competitive OER reaction, overoxidation of alcohols, and corrosion of anode materials.^[^ ^151^ ^]^ In addition, the extra purification and extraction of soluble target products are required.^[^ ^152^ ^]^ In view of this, Li et al.^[^ ^136^ ^]^ propose an all‐solid proton generator‐transfer electrolyzer for benzyl alcohol oxidation using pure benzyl alcohol without the addition of water and simultaneous H_2_ production at the cathode side (Figure 5i). In situ ATR‐SEIRAS spectra detect the key intermediates of alkoxide species (−O−CH_2_−) signal at 1373 cm^−1^, accompanied by the observation of C═O signal of benzaldehyde at 1704 cm^−1^ at a high cell voltage (>0.2 V). These results reveal a cascade dehydrogenation pathway to give generate benzaldehyde (Figure 5j,k). Benefiting from the low thermodynamics potential (0.14 V vs RHE) and water‐free alcohol oxidation, the benzyl alcohol oxidation assisted water electrolysis system maintains a low cell voltage of 1.2 V for 10 days and a high Faradaic efficiency of 80−93% (Figure 5l).

**Table 2:** Comparison of various oxidative products of alcohol oxidation reactions and two‐electrode performance coupled with H_2_ production.

Substrate	Product	Catalyst	Electrolyte	Cell Voltage (V@10 mA cm^−2^)	FE (%, anode)	Ref.
**Methanol**	Formate	CeF_3_@Ni_3_N/CC	1.0 M KOH+ 1.0 M CH_3_OH	1.56^a)^	90	[153]
**Methanol**	CO_2_	Pt_1.8_Pd_0.2_CuGa/C	1.0 M KOH+ 1.0 M CH_3_OH	0.552	–	[118]
**Methanol**	CO_2_	Co‐Rh_2_	1.0 M KOH+ 1.0 M CH_3_OH	1.545	–	[117]
**Methanol**	CO_2_	Cu_2_O‐Cu@Ni_2_P/NF	1.0 M KOH+ 1.0 M CH_3_OH	1.40	–	[126]
**Methanol**	Formic acid	Ni_3_S_2_/CNTs	1.0 M KOH+ 1.0 M CH_3_OH	1.50^a)^	95	[128]
**Methanol**	Formic acid	NiIr‐MOF/NF	1.0 M KOH+ 4.0 M CH_3_OH	1.56	≈100	[119]
**Methanol**	Formic acid	Ni_50_Co_15_Fe_30_Cu_5_ MEAAs	1.0 M KOH+ 1.0 M CH_3_OH	1.649	–	[154]
**Methanol**	Formic acid	NiMnLDH	1.0 M KOH+ 3.0 M CH_3_OH	1.33	≈100	[111]
**Ethanol**	Acetic acid	o‐c‐CoSe_2_‐Ni	1.0 M KOH+ 1.0 M EtOH	≈1.3	≈94	[134]
**Ethanol**	Acetic acid	m‐PtPb NSs	1.0 M KOH+ 1.0 M EtOH	–	90	[65]
**Ethanol**	Acetic acid	NiOOH‐CuO	1.0 M KOH+ 1.0 M EtOH	1.611^b)^	–	[129]
**Ethanol**	Acetic acid	PtCu NF/C	1.0 M KOH+ 0.5 M EtOH	0.58	98.2	[135]
**Ethanol**	Acetic acid	Pd_2_Ga/C	0.5 M KOH+ 0.5 M EtOH	0.62	–	[133]
**Ethanol**	Ethyl Acetate	Co_3_O_4_ NSs	1.0 M KOH+ 1.0 M EtOH	–	98	[155]
**Ethanol**	Sodium acetate	Pd/TNTA‐web	2.0 M KOH+ 2.0 M EtOH	1.76^c)^	–	[156]
**Ethanol**	1,1‐Diethoxyethane	PtIr NWs	0.5 M H_2_SO_4_+ EtOH	0.61	85	[157]
**Glycerol**	Formic acid	NiS_x_/Ni NRAs	0.5 M H_2_SO_4_+ 0.1 M glycerol	–	63.9	[158]
**Glycerol**	Oxalate	Ni‐phen‐NO_2_	2.0 M KOH+ 0.1 M glycerol	1.675^d)^	45.3	[159]
**Glycerol**	Formic acid	Mn‐Co‐S/NF	1.0 M KOH+ 0.1 M glycerol	1.50^d)^	87.8	[160]
**Glycerol**	Formic acid	Ru@MnO_2‐x_	1.0 M KOH+ 0.5 M glycerol	1.68^e)^	92	[161]
**Glycerol**	Formic acid	NiVRu‐LDHs	1.0 M KOH+ 0.1 M glycerol	1.93^c)^	97	[138]
**Glycerol**	Formic acid	MnO_2_/CP	5 mM H_2_SO_4_+ 0.2 M glycerol	1.36	53	[139]
**Glycerol**	Tartronic acid	Au@NiS_x_	0.1 M sodium tetraborate+0.1 M glycerol	–	90.7	[137]
**Glycerol**	Formic acid	HEA‐CoNiCuMnMo	0.1 M KOH+ 0.1 M glycerol	1.63	92	[112]
**Glycerol**	Oxalate	NiO_x_/ MWCNTs‐O_x_	1 M KOH+ 1 M glycerol	–	96	[142]
**Glycerol**	Glycerate	MoO_x_/Pt	1 M KOH+ 0.1 M glycerol	0.70	–	[143]
**Waste polymer‐derived Ethylene** **glycol**	Glycollic acid	(PtIr)(FeMoBi)	1 M KOH+ 1 M EG	0.48	95	[162]

^a)^ Current Density (100 mA cm^−2^);

^b)^ Current Density (50 mA cm^−2^);

^c)^ Current Density (1 A cm^−2^);

^d)^ Current Density (20 mA cm^−2^);

^e)^ Current Density (500 mA cm^−2^)

##### Electrooxidation Synthesis of Azo Energetic Compounds

Energetic materials (EMs) are key components in applications such as aerospace, mining, demolition, and tunneling, which can be classified as explosives, propellants, and pyrotechnics.^[^ ^165^, ^166^ ^]^ Among which, the explosives and propellants requires high‐energy‐density features that are triggered by the friction, impact, spark, or shock to undergo rapid and heat‐producing decomposition. The key for high‐energy density materials (HMDMs) includes C/H/N ratio, density, oxygen balance, heat of formation, etc. The traditional EMs involved these characters are trinitrotoluene (TNT), octogen (HMX), hexogen (RDX), hexanitrohexaazaisowurtzitane (CL‐20), etc. Nevertheless, the majority of these nitro explosives are environmentally unfriendly because of the high pollution of explosive compounds to surface/ground waters, soils, and sediments.^[^ ^166^ ^]^ High‐nitrogen EMs are potential substitutes because they acquires energy from large positive heats of formation via large number of N–N and C–N bonds breakage, rather than by intramolecular oxidation of carbon backbone in traditional EMs. As a result, generation of N_2_ as a main decomposition product of high‐nitrogen EMs is desired to avoid environmental risk. The typical high‐nitrogen EMs such as triazoles, tetrazole, and triazines, etc., have been used to develop new EMs with high‐ performance and low sensitivity.^[^ ^167^ ^]^ However, traditional organically synthetic methodologies usually use hazardous reagents, produced waste streams, and worked under high‐temperature condition. In addition, another purification step is required to separate by‐products, which is uneconomical and harmful to environment.^[^ ^168^ ^]^

Organic electrochemistry has attracted much attention and offers many advantages over traditional pathway such as high reactivity, high stereoselectivity, cost‐effectiveness, decreased environmental toxicity, and inherent scalability.^[^ ^169^, ^170^ ^]^ The electron or electro‐stimulated radical in electrosynthesis can be green substitutes for traditional radical reaction, thus eliminating harmful redox reagents.^[^ ^171^ ^]^ However, there is a notable lack of development on the electrochemical synthesis of EMs. To meet the aims of sustainable and atom‐economical strategies, some pioneer works regarding to the green electrochemical synthesis of EMs have been presented.^[^ ^18^, ^172^, ^173^, ^174^, ^175^, ^176^ ^]^ Sheremetev et al.^[^ ^18^ ^]^ introduce the green electrooxidation of aminofurazans to various azofurazans EMs using NiOOH as the anodic catalyst. As a result, 3‐amino‐4‐methylfurazan, 4,4′‐dimethylazofurazan, 4,4′‐diethylazofurazan, 4,4′‐dipropylazofurazan, etc., energetic compounds are successfully electro‐synthesized in ≈1% aqueous alkali. Afterward, Petrosyan et al.^[^ ^174^ ^]^ report electrochemical N–N coupled of aminofuroxans to azofuroxans that is driven by the electrogenerated NaOCl, NaOBr, and NiO(OH). The electrosynthesized azofuroxan show high conversion and yield of 95% and 80%, respectively, revealing a quite promising for the HMDMs. He et al.^[^ ^176^ ^]^ firstly report the synthesis of 5,5′‐azotetrazolate (ZT) salts by photoelectrochemical pathway. The W, Mo‐BiVO_4_ is used as the catalysts benefitting from the higher standard electrode potential of BiVO_4_ (2.4 V vs RHE) than that of traditional KMnO_4_ oxidant (1.679 V vs RHE). The higher standard electrode potential presents the stronger oxidizability. The BiVO_4_ photoanode can capture solar energy and drive photo‐electrooxidation of 5‐amino‐1*H*‐tetrazole (5AT) to produce ZT EMs (5ATOR) at room temperature with the faradaic efficiency higher than 80% at the constant potential of 1.2 V versus RHE. After that, the graphitic C_3_N_4_ (g‐C_3_N_4_) and Ti doped Fe_2_O_3_ film photoanodes are used in the photoelectrochemical synthesis of ZT EMs.^[^ ^177^, ^178^ ^]^ Based on the above experiences, they introduce the electrochemical synthesis of ZT on various electrodes such as Pt, Fe, Co, Ni, Ti, Mo, C, etc., in Na_2_CO_3_ aqueous solution, and finds that the feasibility of oxidative‐coupled of 5AT into ZT EMs depended on the type of electrode. The 5AT oxidation mechanism of typical Ni and Pt are also proposed. The deprotonation reaction can be appeared on both electrodes. The obtained 5AT^−^is directly coupled to form ZT on Ni electrode, while the •OH formed by OER to oxidize amino group of 5AT^−^ and coupled with another 5AT^−^ to produce ZT EMs.^[^ ^173^ ^]^ Considering the great potential of green electrosynthesis of value‐added EMs, it is possible to couple H_2_ production with electrosynthesis of EMs since some of these reaction showed lower thermodynamic potential than that of OER.^[^ ^176^ ^]^

**Figure 6:**
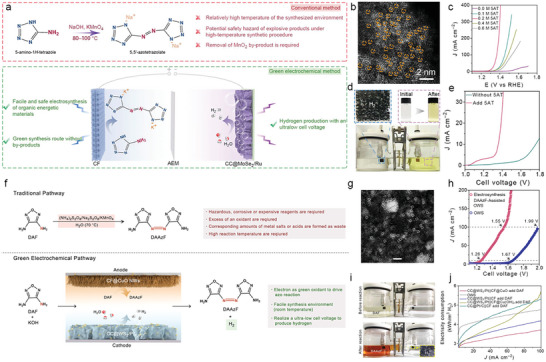
a) Schematic illustration of conventional and green electrosynthesis of PZT EMs. b) AC HAADF‐STEM image of CC@MoSe_2_/Ru SAs. c) LSV curves of 5ATOR under different 5AT concentrations. d) Optical photos of the coupled system. e) LSV curves of the two‐electrode cell before and after the addition of 5AT. Reproduced with permission.^[^ ^163^ ^]^ Copyright 2022. Wiley‐VCH. f) Schematic illustration of conventional and green electrosynthesis of DAAzF EMs. g) HAADF‐STEM image of WS_2_/Pt_1,n_. h) LSV curves of the two‐electrode cell with or without the addition of DAF. i) Optical photos of the coupled system. j) Electricity consumption of the coupled system on different catalysts. Reproduced with permission.^[^ ^164^ ^]^ Copyright 2023. Springer Nature.

In view of this, our group firstly propose the coupled system of green electrosynthesis of EMs and H_2_ production.^[^ ^163^, ^164^, ^179^, ^180^, ^181^ ^]^ This coupled system can avoid hazardous synthetic condition of traditional pathway, and realize low‐cell‐voltage H_2_ production simultaneously (**Figure** **6**a). The Ru single atoms supported MoSe_2_ nanosheets on carbon cloth (CC@MoSe_2_/Ru SAs) is prepared by electrodeposition method as cathodic HER electrocatalysts. Aberration‐corrected (AC) TEM image showed the Ru SAs (bright spot) are dispersed uniformly on the surface of MoSe_2_ (Figure 6b). Before the construction of coupled system, the concentration of 5AT toward 5ATOR is investigated on copper foam (Figure 6c). The optimal concentration of 5AT (0.2 M) is then used in coupled system. The CC@MoSe_2_/Ru SAs and copper foam are cathode and anode electrodes, respectively. 1.0 M KOH and 1.0 M KOH+0.2 M 5AT are catholyte and anolyte, respectively (Figure 6d). LSV curves show that the cell voltage of H_2_ production is reduced to 1.35 V compared to conventional OWS (1.76 V), 410 mV potential is saved, realizing the low‐energy‐consumption H_2_ production. Meanwhile, the color of anolyte is changed from transparent to yellow, indicating the formation of EMs (Figure 6d,e). NMR, UV–vis, and FTIR prove the success formation of ZT EMs.^[^ ^163^ ^]^ In addition, the 3,3′‐diamino‐4,4′‐azofurazan (DAAzF) EMs are successfully synthesized from 3,4‐diaminofurazan (DAF) starting materials oxidation reaction (DAFOR) by the green electrochemical pathway (Figure 6f).^[^ ^164^ ^]^ The cathodic materials are in co‐existence of Pt NPs and single‐atoms (Pt_1,n_) on WS_2_ nanosheets, and the anodic materials are CuO NWs (Figure 6g). This value‐added DAFOR reaction is demonstrated to thermodynamically more favorable than OER, thus can drive low‐energy‐consumption hydrogen production. As a consequence, a low cell voltage of 1.26 V can be obtained at 10 mA cm^−2^, much lower than that of OWS (Figure 6h). After the reaction, the color of anodic electrolyte changes to orange, and the H_2_ gas can be observed at the cathodic side (Figure 6i). Electricity consumption calculation reveals that the energy consumption of this coupled system is 3.70 kWh m^−3^ of H_2_, much lower than that of OWS (≈5.0 kWh m^−3^ of H_2_), indicating the energy‐saving feature of the coupled system (Figure 6j).

Recently, Zhao et al. propose the electrochemical production of 3,3′‐(1E)‐1,2‐diazenediylbis[1‐methyl‐1H‐pyrazole] on Ni‐MOF with a low theoretical potential (0.67 V vs RHE).^[^ ^182^ ^]^ The result shows the 18.5‐fold increase in hydrogen production compared with OWS. Mechanism study of in situ FTIR, Raman, and DFT calculations reveal that the Ni sites coordinated with two H_2_O, two bridging lattices O, and two 1,4‐benzenedicarboxylic acid ligands as the real active sites showed more favorable for azo electrolysis.

As another important EMs, copper azide (CuN_3_) is a kind of popular primary explosive to replace lead azide (LA) and lead styphnate (LS), which are usually suffered from high electrostatic sensitivity resulting from the LS component, and led to many terrible explosion accidents through electrostatic discharge.^[^ ^186^ ^]^ Therefore, much efforts have been devoted to preparing CuN_3_ as substitutes because of its higher flame sensitivity and powerful initiation ability than LA and LS. More importantly, Cu is less toxic than lead and the replacement of CuN_3_ to LA/LS can alleviate risk to both the human health and environment.^[^ ^187^ ^]^ However, the traditional gas–solid azidation method for preparing CuN_3_ usually involves high‐toxic, explosive, severely corrosive, and readily self‐ignitable of HN_3_ as nitrogen source. What's more, the insufficient gas–solid azidation reaction is often long‐term (>12 h) and the remaining HN_3_ requires extra postprocessing process.^[^ ^188^ ^]^ The green and sustainable electrochemical pathway to prepare CuN_3_ is the “savior” for refraining from traditional gas–solid azidation reaction.^[^ ^187^, ^189^, ^190^ ^]^ Zhu et al.^[^ ^187^ ^]^ introduce the NaN_3_ as the nitrogen source to replace hazardous HN_3_ in the electrolyte. A constant current density of 3 mA cm^−2^ is applied for 450 s on porous Cu film. The bure CuN_3_ is successfully obtained by the reaction equations: Cu(solid) – e → Cu^+^; Cu^+^ + N_3_^−^ (aqueous) → CuN_3_ (solid). Considering the relatively low potential of Cu/Cu^+^ (0.521 V vs RHE), it is speculated that the electrochemical azidation reaction may be a potential alternative for OER to couple with H_2_ production. As a result, our group propose the integration of electrosynthesis of CuN_3_ and H_2_ production system.^[^ ^191^ ^]^ The concentration of NaN_3_ toward activity of electrosynthesis of CuN_3_ is investigated under three‐electrode system. The optimal value (0.08 M) is then applied in anolyte of coupled system including 1.0 M Na_2_SO_4_. LSV curves showed that the cell voltage is reduced to 1.07 V compared with conventional OWS (≈1.5 V), indicating the low‐energy‐consumption H_2_ production. The optical photograph of coupled system show the color transformation from red to yellow green, together with the XRD pattern and SEM image proved the successful synthesis of pure CuN_3_ EMs. In addition, the H_2_ bubble is released from CC@MoS_2_/Ru NPs cathode. The above discussion reveals that some of EMs electrosynthesis can be a potential alternative to OER for low‐energy‐consumption H_2_ production. Meanwhile, green and sustainable pathway to prepare EMs can be achieved.

#### 2.2.2 Value‐Added Sacrificial‐Agent Oxidation Reaction

Apart from small molecules oxidation reaction to assist OWS for energy‐saving hydrogen production and value‐added chemicals at anode side, sacrificial‐agent‐assisted OWS are another popular strategy for high‐efficiency hydrogen production such as hydrazine and urea, etc. Researchers find that the electro‐oxidative decomposition of these small molecules required much lower thermodynamic potential than OER. Therefore, the coupled of these electrooxidation reactions with OWS can realize low‐cell‐voltage hydrogen production.^[^ ^64^, ^192^ ^]^ In addition, water pollution is a serious issue from the perspective of environmental protection. Photo‐degradation and advanced oxidation reaction have been utilized for sewage treatment. Especially, electrochemical pathway for degradation of pollutants is appealing due to its advantages such as cost‐effectiveness, mild working conditions, high universality and easy automation.^[^ ^193^ ^]^ Urea and hydrazine are also considered as environmental pollutants in water, the electrooxidation degradation of these pollutants can avoid using extra oxidants and complex separation process.^[^ ^194^ ^]^ This section provides a summary of recent advances in sacrificial‐agent‐assisted OWS using various state‐of‐the‐art electrocatalysts and the corresponding catalytic mechanisms investigation.

##### Hydrazine Oxidation Reaction (HzOR)

**Figure 7:**
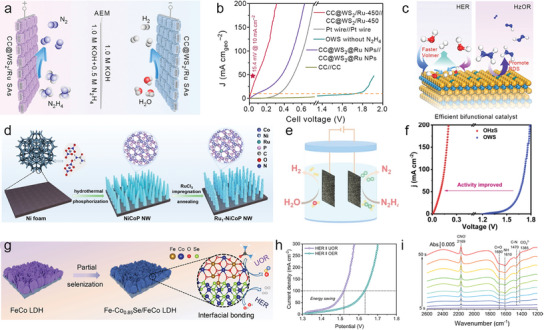
a) Schematic illustration of the coupled system of HzOR‐assisted OWS. b) LSV curves of the HzOR‐assisted OWS using various catalysts. c) Mechanism illustration of the HzOR and HER on CC@WS_2_/Ru‐450 bifunctional catalysts. Reproduced with permission.^[^ ^183^ ^]^ Copyright 2022. Wiley‐VCH. d) The synthesis procedure of Ru_1_NiCoP NWAs. e) Schematic illustration of the coupled system of HzOR‐assisted OWS. f) LSV curves of the HzOR‐assisted OWS and traditional OWS using Ru_1_NiCoP||Ru_1_NiCoP catalysts. Reproduced with permission.^[^ ^184^ ^]^ Copyright 2023. Wiley‐VCH. g) The synthetic process of Fe‐Co_0.85_Se/FeCo LDH. h) LSV curves of the HzOR‐assisted OWS and traditional OWS. i) In situ time‐resolved FTIR at a constant potential of 1.45 V. Reproduced with permission.^[^ ^185^ ^]^ Copyright 2023. Wiley‐VCH.

Hydrazine is one of the important industrial raw materials and has been widely used as corrosion inhibitors, rocket fuels, and deoxidant in the feed water of power plants.^[^ ^194^, ^195^ ^]^ The electrooxidation degradation of hydrazine pollutants in wastewater shows much advantages than traditional pathways (e.g., Fenton's reagent), including the only by‐products of nitrogen gas and water without greenhouse gas and zero‐carbon reaction without the generation of catalyst‐poisoning species. Moreover, the HzOR (N_2_H_4_ + 4OH^−^ → N_2_ + 4H_2_O + 4e^−^, −0.33 V vs RHE) has great potential for producing hydrogen at a lower cell voltage because of the much lower thermodynamic potential of HzOR than that of OER (1.23 V vs RHE), thus the integration of HzOR and HER in coupled water electrolysis system have been widely investigated. Despite the fast development of the HzOR‐assisted OWS, the exploration of highly‐efficient bifunctional electrocatalysts is still a big challenge due to the different reaction mechanism of HER and HzOR. In addition, the working potential of HzOR still far from the theoretical one.^[^ ^196^ ^]^ So far, considerable endeavors have been devoted to exploring advanced electrocatalysts for HzOR. The catalysts such as Rh/Pd metallene,^[^ ^197^ ^]^ RhRu_0.5_,^[^ ^64^ ^]^ NiRh‐MOF,^[^ ^198^ ^]^ Ru‐Ni(OH)_2_,^[^ ^199^ ^]^ Ni SACs/Ti_3_C_2_T_x_,^[^ ^200^ ^]^ FeNiP‐NPHC,^[^ ^201^ ^]^ Ni_3_N‐Co_3_N,^[^ ^57^ ^]^ Ru‐NiFeLDH,^[^ ^202^ ^]^ Pt@NiFc‐MOF,^[^ ^203^ ^]^ Pt@Ni_3_N‐MoN/Ti,^[^ ^204^ ^]^ etc., have been achieved appreciable performance. Recently, our group has introduced bifunctional Ru active sites for promoting alkaline HER and HzOR by lowering the energy barrier of RDS for water dissociation during HER and stepwise dehydrogenation during HzOR.^[^ ^183^ ^]^ As a consequence, the HzOR‐assisted OWS coupled system only acquires 15.4 mV at the current density of 10 mA cm^−2^, which is much higher than that of OWS (1.72 V) (**Figure** **7**a,b). DFT calculations reveal that the Ru SAs sites on WS_2_ substrate are acted as bifunctional active sites for promoting water dissociation in HER and dehydrogenation in HzOR (Figure 7c). We also evaluate the pH‐universal performance of the Ru‐based WO_3_ catalysts and exhibited superior HER and HzOR activity in alkaline and acid media.^[^ ^205^ ^]^

Li et al. report a Ru_1_‐NiCoP single atom catalysts prepared by hydrothermal/phosphorization and RuCl_3_ impregnation/annealing processes (Figure 7d).^[^ ^184^ ^]^ The obtained Ru_1_‐NiCoP as superior bifunctional electrocatalyst toward HzOR‐assisted OWS, shows only 90 mV to reach 50 mA cm^−2^, 1.57 V lower than that of OWS (Figure 7e,f). DFT calculations reveal that the dehydrogenation of *HNNH_2_ to *N_2_H_2_ is RDS for HzOR, and the Ru incorporation in NiCoP can lower energy barrier of RDS by enhancing the adsorption ability toward *N_2_H_2_ intermediate. Different from the traditional stepwise dehydrogenation mechanism of HzOR, Shi et al. propose a new reaction path of N–N single bond breakage that the N_2_H_4_ molecule is firstly adsorbed on the surface of Ni–Co–P/NF to form *NH_2_NH_2_, the N–N bond is then broken and formed two *NH_2_, which is gradually dehydrogenated to generate N_2_.^[^ ^206^ ^]^ For commercial applications, the performance at high current density (500–1000 mA cm^−2^) need to be achieved, which is still a formidable task. Qiu et al.^[^ ^194^ ^]^ report the NiCo@C/MXene/CF self‐standing electrode to realize a low voltage of 0.31 V at a high current density of 500 mA cm^−2^ in alkaline seawater splitting coupled with HzOR and 0.42 V at 400 mA cm^−2^ in neutral seawater. This activity is still far excels the OWS and avoid chlorine evolution reaction (ClOR, *E*° = 1.36 V).^[^ ^207^ ^]^ The electricity input for the coupled seawater is as low as 2.75 kWh m^−3^ H_2_ at 500 mA cm^−2^, 48% decrease in energy equivalent input relative to commercial OWS.

##### Urea Oxidation Reaction (UOR)

**Figure 8:**
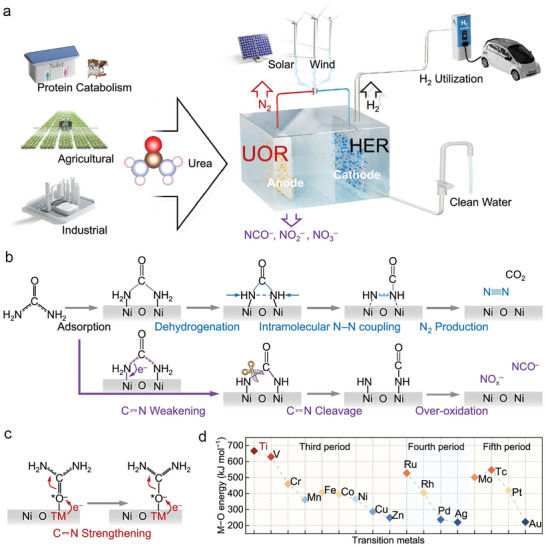
a) Conception of UOR for clean hydrogen production. b) Two UOR pathways on nickel and its oxides (NiO, NiOOH) substrate. c) The strategy of strengthening C⎓N bond by the electron transfer from substrate TM atoms to the adjacent O moiety. d) The bond formation energies of O with various TMs. Reproduced with permission from.^[^ ^208^ ^]^ Copyright 2024. Springer Nature.

As another sacrificial‐agent oxidation reaction, electrochemical urea oxidation reaction (UOR) can be used in both powering fuel cells and alternatives to OER for energy‐saving OWS because of the six electron–proton coupled transfer step (CO(NH_2_)_2_ + 6OH^−^ → N_2_ + CO_2_ + 5H_2_O + 6e^−^) with a lower thermodynamic potential of 0.37 V versus RHE than that of OER.^[^ ^192^, ^209^, ^210^, ^211^ ^]^ In addition, urea is present in waste water, human urine, and by‐product of industrial activities, which can be greenly degraded through UOR (**Figure** **8**a). Moreover, the UOR‐assisted OWS can also avoid chlorine gas generation in seawater splitting due to the lower thermodynamic potential (1.36 V). There are some recent works focus on advanced electrocatalysts design for UOR||HER such as RuCu,^[^ ^212^ ^]^ V‐Co_2_P_4_O_12_,^[^ ^213^ ^]^ NiCoCr‐LDH,^[^ ^45^ ^]^ CoS_1.097_/Ni_3_S_2_,^[^ ^214^ ^]^ F‐NiO/Ni@C,^[^ ^215^ ^]^ Mo‐NiS,^[^ ^216^ ^]^ Ni_3_N/Mo_2_N,^[^ ^217^ ^]^ Ni_2_P‐Co_2_P/C,^[^ ^58^ ^]^ NiCoMoCuO_x_H_y_,^[^ ^218^ ^]^ etc. However, at present, the working potential of UOR is still larger than 1 V versus RHE that is attributed to the inherent 6 electron‐proton coupled transfer process.^[^ ^58^, ^215^, ^217^, ^219^, ^220^ ^]^ Peng et al.^[^ ^185^ ^]^ introduce Fe‐Co_0.85_Se/FeCo LDH array as bifunctional self‐standing electrode for UOR and HER (Figure 7g). The integrated water electrolysis system shows low cell voltages of 1.32, 1.52, and 1.57 V to achieve 10, 100, and 300 mA cm^−2^, respectively (Figure 7h). DFT calculations reveal that the interfaces in Fe‐Co_0.85_Se/FeCo LDH lowered the free energy change to dissociate CO(NH_2_)_2_* to NCO* and NH* intermediate and enhance the thermodynamic behavior of UOR. The in situ FTIR indicate the possible reaction mechanisms of UOR that involves incomplete oxidative UOR (Figure 7i). However, the widely study of UOR mechanism on Ni based materials involves stepwise dehydrogenation of *CO(NH_2_)_2_ to form *COO. The desorption of *COO to form CO_3_^2‐−^ is RDS for UOR, but the strong binding of the Ni^3+^ active sites with *COO decreases the activity of UOR.^[^ ^192^ ^]^

In view of this, Qiao et al.^[^ ^192^ ^]^ introduce a prussian blue analogs of Ni_2_Fe(CN)_6_ electrocatalyst and delivers one of the best UOR‐assisted OWS activities of 1.38 and 1.50 V to obtain the current density of 10 and 100 mA cm^−2^. Different from the currently understood mechanism, the in situ Raman and DFT calculations reveal that the UOR on Ni_2_Fe(CN)_6_ involved two‐stage thermodynamically favorable process, is that the transformation of urea to NH_3_ and CO_2_ on Ni sites and the Fe sites are responsible for the conversion of NH_3_ to N_2_. It is noted that Ni‐based catalysts usually convert urea to hazardous cyanate (NCO^−^) and nitrite/nitrate (NO^−^) species with a low N_2_ selectivity of <55%, which may poison the anode, decrease the stability of electrolysis system, and be harmful to environment.^[^ ^208^, ^221^ ^]^ The prevalent Ni‐based catalysts with Ni‐O‐Ni sites bond to urea via electron‐donating NH_2_ ends, which blocks the interaction of NH_2_ and C═O groups and weakens the C⎓N. This is the main reason to produce NCO^−^ and NO^−^ species (Figure 8b). Hence, how to avoid the cleavage C⎓N bonds and targeted transformation of urea to N_2_ is the main strategy for highly‐efficient and environmental‐friendly UOR. In view of this, Zhang et al.^[^ ^208^ ^]^ propose the increase the stability of C⎓N bonds by donating electrons to the terminal O of C═O bonds (Figure 8c). The accumulation of electrons around C⎓N can be achieved by pairing Ni sites with pronounced oxygenophilic affinity. They then assess the periodic table and found that Ti is the optimal alternative to Ni due to its higher bond formation energy (667 kJ mol^−1^) with O than that of Ni (366 kJ mol^−1^) and other TMs (Figure 8d). The elaborate Ni–O–Ti sites are then constructed by doping Ni SAs on Ti foam with a N_2_ selectivity of 99% for the UOR. In addition, the inhibition of producing NCO^−^ and NO^−^ over 10 days. The constructed solar‐powered UOR–HER device has a potential to process urine in underdeveloped areas of on‐site urine processing. This process can also reduce nitrogen pollution and meanwhile realize clean energy production. The comparison of recently reported sacrificial‐agent oxidation reaction and two‐electrode performance coupled with H_2_ production are collected and listed in **Table** **3**.

**Table 3:** Comparison of recently reported sacrificial‐agent oxidation reaction and two‐electrode performance coupled with H_2_ production.

Substrate	Catalyst	Electrolyte	Oxidative potential (mV vs RHE@10 mA cm^−2^)	Cell voltage (V@10 mA cm^−2^)	Ref.
**Hydrazine**	*a*‐RuMo/NiMoO_4_/NF	1.0 M KOH + 0.5 M N_2_H_4_	−91	0.007	[68]
**Hydrazine**	NiO/Ru	1.0 M KOH + 0.5 M N_2_H_4_	−79	0.021	[62]
**Hydrazine**	NiRu‐ABDC	1.0 M KOH + 0.3 M N_2_H_4_	−83	0.029	[50]
**Hydrazine**	*α*‐MoC/N‐C/Ru_NSA_	1.0 M KOH + 0.3 M N_2_H_4_	−80	0.064	[224]
**Hydrazine**	FeOOH/Ni_12_P_5_/Ni_2_P	1.0 M KOH + Seawater + 0.4 M N_2_H_4_	−8	0.22	[225]
**Hydrazine**	Pt@NiFc‐MOF	1.0 M KOH + 0.5 M N_2_H_4_	357^a)^	0.518^a)^	[203]
**Hydrazine**	Ru‐O‐Ni/Fe	1.0 M KOH + 0.3 M N_2_H_4_	−75	0.43^b)^	[202]
**Hydrazine**	Mn@Ni_3_N‐Co_3_N/NF	1.0 M KOH + 0.5 M N_2_H_4_	≈–100^c)^	0.49^c)^	[57]
**Hydrazine**	Pt QDs@Ni_3_N‐MoN/Ti	1.0 M KOH + Seawater + 0.5 M N_2_H_4_	−11	1.12^b)^	[204]
**Hydrazine**	Ru_1_‐NiCoP	1.0 M KOH + 0.3 M N_2_H_4_	−60	0.09^d)^	[184]
**Hydrazine**	NiRh‐MOF	1.0 M KOH + 0.3 M N_2_H_4_	17	0.06	[198]
**Hydrazine**	Ru‐Ni(OH)_2_ NW^2^/NF	1.0 M KOH + 0.1 M N_2_H_4_	−79^e)^	152^e)^	[199]
**Hydrazine**	Ni‐Co‐P/NF	1.0 M KOH + 0.1 M N_2_H_4_	−61	0.88^f)^	[206]
**Hydrazine**	RhRu_0.5_	1.0 M KOH + 0.1 M N_2_H_4_	≈–20	0.054^e)^	[64]
**Hydrazine**	Co(OH)_2_/MoS_2_/CC	1.0 M KOH + 0.4 M N_2_H_4_	177^e)^	0.142	[226]
**Hydrazine**	N‐Ni_5_P_4_@CoP/CFP	1.0 M KOH + 0.1 M N_2_H_4_	−32	0.037	[227]
**Hydrazine**	Ru‐(Ni/Fe)C_2_O_4_	1.0 M KOH + 0.1 M N_2_H_4_	−96	0.01	[228]
**Hydrazine**	Ni SACs/Ti_3_C_2_T_x_	1.0 M KOH + 0.1 M N_2_H_4_	−30	−	[200]
**Hydrazine**	FeNiP‐NPHC	1.0 M KOH + 0.5 M N_2_H_4_	7^e)^	≈0.3^e)^	[201]
**Urea**	Ni‐In_2_O_3_	1.0 M KOH + 0.33 M Urea	1.31^i)^	−	[44]
**Urea**	NiFe(OH)_x_/ACE	1.0 M KOH + 0.33 M Urea	1.34 (V)	1.412 (V)	[229]
**Urea**	Ni–O–Ti	1.0 M KOH + 0.33 M Urea	1.30 (V)	−	[208]
**Urea**	CoS_1.097_/Ni_3_S_2_	1.0 M KOH + 0.33 M Urea	1.18 (V)	1.33 (V)	[214]
**Urea**	NiCoCr‐LDH/NF	1.0 M KOH + 0.5 M Urea	1.38 (V)	1.427 (V)	[45]
**Urea**	V‐Co_2_P_4_O_12_	1.0 M KOH + 0.5 M Urea	316 ^h)^	1.42 (V)	[213]
**Urea**	RuCu	1.0 M KOH + 0.5 M Urea	1.34 (V)	1.522 (V)	[212]
**Urea**	Ni‐SO_X_	1.0 M KOH + 0.33 M Urea	≈1.37 (V)	−	[230]
**Urea**	Ni_2_P‐Co_2_P/C	1.0 M KOH + 0.33 M Urea	1.27 (V)	−	[58]
**Urea**	F‐NiO/Ni@C	1.0 M KOH + 0.33 M Urea	1.31 (V)	1.37 (V)	[215]
**Urea**	NiCoMoCuO_x_H_y_	1.0 M KOH + 0.33 M Urea	1.32 (V)	1.50^e)^ (V)	[218]
**Urea**	Ru‐Co DAS/NiO	1.0 M KOH + 0.33 M Urea	1.288 (V)	−	[219]
**Urea**	Ni_3_N/Mo_2_N	1.0 M KOH + 0.33 M Urea	1.36 (V)^e)^	1.36 (V)	[217]
**Urea**	Mo‐NiS/CFP	1.0 M KOH + 0.5 M Urea	1.355 (V)^e)^	1.64 (V)^e)^	[216]
**Urea**	N‐Co_9_S_8_/Ni_3_S_2_/NF	1.0 M KOH + 0.5 M Urea	1.41 (V)^f)^	1.40 (V)	[231]
**Urea**	*c*‐CoNiP_x_/a‐P‐MnO_y_	1.0 M KOH + 0.5 M Urea	1.24 (V)	≈1.67 (V)	[232]

^a)^ Current density (1500 mA cm^−2^);

^b)^ Current density (1 A cm^−2^);

^c)^ Current density (500 mA cm^−2^);

^d)^ Current density (50 mA cm^−2^);

^e)^ Current density (100 mA cm^−2^);

^f)^ Current density (200 mA cm^−2^);

^g)^ Onset potential;

^h)^ Overpotential;

^i)^ Current density (25 mA cm^−2^).

#### 2.2.3 Other Value‐Added Oxidation Reaction

##### Sulfion oxidation

**Figure 9:**
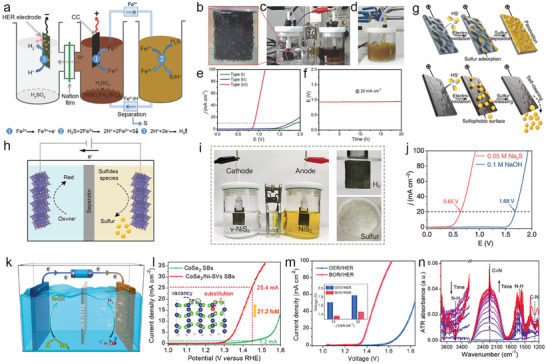
a) Concept of the coupled systems of sulfur collection and HER. b–d) Photograph of the coupled system. e) LSV curves of CC@N‐CoP//CC (type I), CC@N‐CoP//Pt (type II), and CC@N‐CoP//CC (type III) in 0.5 M H_2_SO_4_. f) CP test of the coupled system. Reproduced with permission.^[^ ^51^ ^]^ Copyright 2018. Wiley‐VCH. g) Passivated electrodes by the sulfur generation during long‐term operation. h,i) Schematic illustration and photograph of the sulfion oxidation‐assisted OWS coupled system. j) LSV curves of the coupled system and conventional OWS. Reproduced with permission.^[^ ^222^ ^]^ Copyright 2021. Wiley‐VCH. k) Schematic illustration of amine oxidation‐assisted OWS. l) LSV curves of benzylamine electrooxidation (BOR) on CoSe_2_ and CoSe_2_/Ni‐SVs. m) LSV curves of the BOR‐assisted OWS and conventional OWS. n) in situ ATR FTIR spectra of benzylamine electrooxidation on CoSe_2_/Ni‐SVs with 0.02 M butylamine in 0.1 M KOH at the potential of 0.45 V versus RHE for 30 min. Reproduced with permission.^[^ ^223^ ^]^ Copyright 2022. American Chemistry Society.

Hydrogen sulfide (H_2_S) or S^2‐−^, as a highly undesirable contaminant, is existed ubiquitously in natural gas resources, industrial wastewaters from paper mill, tannery, and petrochemical plants.^[^ ^51^, ^222^ ^]^ The electrochemical sulfion oxidation reaction (SOR) as an alternative sulfide abatement technology is able to remove toxic, malodorous and corrosive H_2_S compounds or S^2−^ from the above high concentration H_2_S gas fields and help address serious environmental pollution. Moreover, the relatively low intrinsic thermodynamic equilibrium potential of SOR (S^2−^ − 2e^−^ → S, *E_0_* = –0.48 V versus RHE; HS^−^ + OH^−^ → S + H_2_O + 2e^−^, *E_0_* = 0.142 V vs RHE) can dramatically reduce applied cell voltage of OWS and avoid poisonous chlorine chemistry because of the much higher thermodynamic equilibrium potential of ClOR.^[^ ^233^, ^234^ ^]^ If the electrochemical absorption and conversion of H_2_S are able to replace OER and couple into a highly effective OWS for H_2_ production, the OWS will become even more economically viable. Zhang et al.^[^ ^51^ ^]^ introduce the electrochemical SOR‐coupled OWS mediated by Fe^3+^/Fe^2+^ redox (**Figure** **9**a). When the HER occurs on the cathodic side (N‐CoP as the high‐efficiency catalysts), Fe^2+^ is oxidized to Fe^3+^ on the counter side. Such process is reversible by involving SOR in the absorption reactor to provide proton for cathodic HER and sulfur collection (Fe^3+^ + H_2_S = Fe^2+^ + S↓ + 2H^+^) (Figure 9b–d). The novel coupled system delivers ultralow cell voltage of 0.89 V to achieve 10 mA cm^−2^ on CC@N‐CoP//CC in 0.5 M H_2_SO_4_, much lower than those of CC@N‐CoP//Pt (1.76 V) and conventional OWS on CC@N‐CoP//CC (1.91 V) (Figure 9e). The energy consumption decreases by 53% compared to OWS, revealing energy‐saving hydrogen production and concurrently recover of the useful sulfur resources. The coupled system also shows robust stability for 20 h at 20 mA cm^−2^ (Figure 9f). However, the accumulate deposited sulfur on the electrode will passivate the electrode surface, limit the mass transfer, raise the energy consumption, and make continuous operation infeasible.^[^ ^235^ ^]^ Some traditional technologies such as mechanically rotate electrode, dissolving sulfur with organic solvents, and using redox mediators have been introduced to remove sulfur from passivated electrode.^[^ ^236^, ^237^ ^]^

Despite these efforts, the indirect strategy for activating electrode will increase energy consumption and cost of the whole process. In view of this, Zhang et al.^[^ ^222^ ^]^ introduce a self‐cleaning NiS_2_ electrode with a low affinity to elemental sulfur feature and show de‐wetting properties of electrode, which enables long‐term self‐cleaning desulfurization during SOR (Figure 9g). They also design NiS_2_ with abundant S vacancies (*v*‐NiS_2_) for more thermoneutral H* adsorption. As the bifunctional catalysts, the NiS_2_ and v‐NiS_2_ are used as the anode and cathode catalysts respectively in SOR‐assisted OWS (Figure 9h). The result shows that the anode is not passivated by sulfur and H_2_ is evolved concurrently (Figure 9i). LSV curves exhibit the lower cell voltage of 0.65 V in 0.05 M Na_2_S+0.1 M NaOH compared to that of conventional OWS (1.68 V) to achieve 20 mA cm^−2^ (Figure 9j). Such a strategy not only realizes energy‐saving hydrogen production, but also achieves continuous SOR to generate sulfur so that the environmental pollution induced by H_2_S can be inhibited. Considering the practical application requirements, the operation at higher current density (at least 100 mA cm^−2^) should be taken into account.^[^ ^238^ ^]^ Yang et al.^[^ ^239^ ^]^ prepare Co_3_S_4_ nanowires on nickel foam as bifunctional catalysts for SOR‐assisted seawater splitting. Benefiting from the metallic nature, superior electrocatalytic activity, moderate binding energy, and unique morphology, the as‐prepared catalysts show lower cell voltages of 0.496 V and 0.608 V to reach 100 and 300 mA cm^−2^, 1.289 V decreased than that of conventional seawater splitting (1.785 V at 100 mA cm^−2^). As a result, a low electricity input of only 1.185 kWh m^−3^ H_2_ at 300 mA cm^−2^ is obtained, saving over 70% of the electricity compared with that of conventional OWS (>4.5 kWh m^−3^ H_2_). DFT calculations reveal that the S^2−^ is adsorbed spontaneously on the surface of Co_3_S_4_ and reduces the energy barrier of RDS (S_3_^2−^ → S_4_^−^), which promotes the conversion of polysulfides on the electrode for accelerating SOR.

In addition to the requirement of eliminating corrosion and passivation effects of insulative sulfur products, and meeting the industrial‐scale current densities, the subsequent acidification of anodic polysulfide‐ion products are required to recover the S_8_ elementary sulfur from the disproportionation of S_n_^2‐−^ at relatively low pH conditions.^[^ ^234^ ^]^ However, H_2_S regeneration and acid supplementation are involved in this process, and the traditional anodic elementary sulfur products is not so valuable for practical applications. Therefore, the transformation of polysulfide products to value‐added chemicals is highly desirable at this stage. Zhang et al.^[^ ^240^ ^]^ design a CuCoNiMnCrS_x_ nanosheet high‐entropy sulfide (HES) for SOR process. The synergistical improvements of adsorption of reactant and desorption of insulative sulfur products are achieved by the introduction of a series of harder‐acid cations to accumulate electrons at the Cu^+^ sites, along with the soft‐hard repulsion effect created by the sulfophobic interfaces. As a result, an ultralow cell voltage of 0.73 V is obtained to reach 200 mA cm^−2^, 1130 mV potential lower than that of OWS. At the anodic side, value‐added thiosulfates ($ 37.2–55.8 kmol_Sulfur_^−1^) are generated, compared with the traditional elemental sulfur ($ 1.6–4.8 kmol_Sulfur_^−1^). This novel SOR‐assisted OWS system provides a promising route for sulfur‐containing sewage treatment.

##### Amine Oxidation

Primary amines (R‐NH_2_) oxidation holds crucial role in the synthetic chemistry because of the wide applications of its products including nitriles, imines, amides, azo compounds, and amine oxides, etc.^[^ ^241^ ^]^ Typically, the nitriles have acquired more respect for essential building blocks and intermediates in pharmaceuticals, agrochemicals, and fine chemicals, etc.^[^ ^242^, ^243^ ^]^ However, the harmful HCN or metal cyanides are often used in the synthesis of nitriles through the classic Sandmeyer reaction or the nucleophilic substitutions of aryl and alkyl halides, which hinders the practical applications due to the environmental/safe concerns.^[^ ^244^ ^]^ Despite much efforts have been devoted to develop alternative strategy to the synthesize nitriles, the oxidants (e.g., TEMPO), high pressure, or elevated temperature are necessary. Electrochemical amine oxidation reaction (AOR) provides an aqueous, oxidant‐free, facile and safe synthetic condition. More important, the theoretical oxidation potentials of AOR ranges from 0.5 to 0.8 V versus RHE, which delivers favorable thermodynamic kinetics than OER.^[^ ^245^ ^]^ Therefore, the integration of AOR and OWS to restrain OER is beneficial to rationally reduce the cell voltage and energy consumption of hydrogen production.

Meanwhile, the value‐added products from amine can be realized from green electrochemical AOR. Zhang et al.^[^ ^246^ ^]^ demonstrate that the benzylamine can be a model candidate for AOR on NiSe nanorod arrays electrode. After the introduction of 1 mmol benzylamine, the onset oxidation potential decreased from 1.48 V (OER) to 1.34 V versus RHE, demonstrating the more thermodynamically favorable of AOR than OER. The potential‐dependent Raman reveals that the in situ formed high‐valenced Ni^II^/Ni^III^ are real active centers to convert primary amines to nitriles. Two‐electrode coupled system shows that the cell voltage is as low as 1.49 V compared with conventional OWS (1.70 V) to meet the required current density of 20 mA cm^−2^, indicating a lower energy‐consumption of HER coupled with benzylamine oxidation. Afterword, the high‐efficiency catalysts such as Ni‐Ni_3_N,^[^ ^247^ ^]^ Ni_2_Si,^[^ ^248^ ^]^ Ni(OH)_2_,^[^ ^249^ ^]^ Ni_2_P‐UNMs/NF,^[^ ^250^ ^]^ etc., have been demonstrated superior activity and selectivity to AOR. However, the conversion efficiency optimization or catalytic conversion processes of AOR are not fully explored as it is still at infant stage. In addition, the reported onset potentials of AOR are >1.36 V in alkaline solutions on state‐of‐the‐art AOR catalysts, which is not able to provide a significant reduction of potential compared with state‐of‐the‐art OER activity (≈1.40 V vs RHE). This may reduce the faradic efficiency and selectivity of AOR because of the competitive reaction of OER.

**Figure 10:**
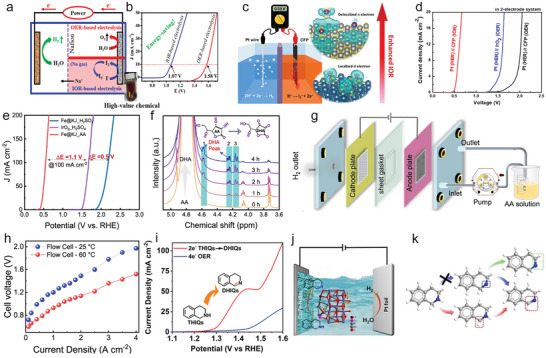
a) Conception scheme and b) LSV curves of IOR‐assisted OWS. Reproduced with permission.^[^ ^252^ ^]^ Copyright 2021. American Chemistry Society. c) Concept of the coupled systems of IOR‐assisted OWS (*inset*: delocalized and localized π electrons of the graphite‐based catalysts). d) LSV curves of the coupled system on various catalysts. Reproduced with permission.^[^ ^253^ ^]^ Copyright 2022. Royal Society of Chemistry. e) LSV curves of Fe@KJ and commercial IrO_2_ in H_2_SO_4_ with or without the addition of ascorbic acid (AA). f) NMR spectra at different time in 1 M AA. g) Schematic diagram of the flow cell. h) LSV curves of flow cell at 25 and 60 °C. Reproduced with permission.^[^ ^254^ ^]^ Copyright 2023. Springer Nature. i) LSV curves of CoFe‐NiSe_2_. j) The coupled system of THIQs oxidation‐assisted OWS. k) The possible mechanism of THIQs oxidation. Reproduced with permission.^[^ ^255^ ^]^ Copyright 2023. Wiley‐VCH.

Guo et al.^[^ ^223^ ^]^ report a Se vacancies and Ni substitutions regulated CoSe_2_ sub‐nanometer belts for boosting AOR and HER (Figure 9k). The elaborate catalysts show ultralow onset potential of 1.30 V versus RHE for electrooxidation of butylamine butyronitrile with a high selectivity of 98.5% (Figure 9l). The assembled two‐electrode coupled system exhibit only 1.37 and 1.47 V to reach 10 and 50 mA cm^−2^, respectively, 320 and 360 mV lower than those of conventional OWS (Figure 9m). In situ FTIR reveal the effective adsorption and consumption of butylamine as evidenced by the weakened intensity of N−H (≈3490 and ∼1616 cm^−1^) and C–N (≈1295 cm^−1^) bonds followed by the increased intensity of C≡N (≈2253 cm^−1^) bond as the electrolysis time increases (**Figure** **10**n). DFT calculations further reveal that Se vacancies are core active sites for N atoms, while the Ni substitution promotes faster stepwise dehydrogenation. Recently, Zhao et al.^[^ ^245^ ^]^ construct a self‐supporting Fe‐Ni_3_S_2_ to promote benzylamine oxidation. LSV curves show that the onset potential decreased from ≈1.5 to ≈1.3 V versus RHE with the addition of 10 mM benzylamine in 1.0 M KOH, confirming the thermodynamically favorable electrooxidation of benzylamine. DFT calculation combined with in situ Raman spectra reveal that the high‐efficiency benzylamine oxidation originated from Fe doping and lattice electrophilic oxygen to lower the energy barrier of RDS. These findings signify the electrochemical oxidation of amines to value‐added chemicals are promising anodic substrates to be applied in high‐efficiency water electrolysis system. After that, they report NiO/NC for electrooxidation of amines to nitriles.^[^ ^251^ ^]^ The onset potential is as low as 1.32 V versus RHE. The in situ spectroscopy and DFT calculations reveal that the electron interaction between NiO and NC induces the positively charge on the surface, favoring adsorption of amine and activating N─H/C─H bond. As a result, a high conversion and selectivity of nitrile (≈99%) are realized; meanwhile, high‐rate hydrogen production (16.4 times higher than OWS) is obtained.

##### Iodide Oxidation

Iodide oxidation reaction (IOR, 2I^−^_(aq)_ → I_2_ + 2e^−^) has a relatively low equilibrium potential of 0.54 V versus RHE compared with OER and its oxidation products of iodine have a wide applications including chemical synthesis,^[^ ^256^ ^]^ food supplements,^[^ ^257^ ^]^ pharmaceuticals,^[^ ^258^ ^]^ medicine, spectroscopy, etc. Therefore, the IOR integrated with OWS enables reduction of energy‐consumption of hydrogen production via decreasing the cell voltage of OWS. Meanwhile, the value‐added commodity chemical of iodine (I_2_) rather than O_2_ at the anode side is obtained.^[^ ^252^, ^253^, ^259^, ^260^ ^]^ However, the IOR‐assisted OWS strategy has been given little to no attention in energy conversion sectors. Hwang et al.^[^ ^252^ ^]^ first propose the IOR‐assisted OWS on ruthenium–tin surface alloy oxide (RuSn SAO) catalysts for promoting IOR (Figure 10a), which shows higher activity of only 1.01 V versus RHE at 10 mA cm^−2^ under acid medium (0.1 M HClO_4_) than that of neutral medium (1.22 V vs RHE). The coupled system is then conducted in H‐type two‐electrode system with 0.1 M HClO_4_ and 0.1 M HClO_4_+0.1 M NaI as cathodic and anodic electrolytes, respectively. The result shows that an ultralow cell voltage of 1.07 V is obtained to achieve 10 mA cm^−2^, which is 510 mV lower than that of conventional OWS (Figure 10b), thus realizing the low‐energy‐consumption hydrogen production and obtaining value‐added I_2_ chemicals as well as eliminating the drawbacks brought by OER such as explosive risk of H_2_/O_2_ gas mixing and formation of ROS. After that, the high‐efficiency catalysts such as Ni‐Co(OH)_2_,^[^ ^259^ ^]^ RuTiO,^[^ ^260^ ^]^ and CFP,^[^ ^253^ ^]^ are applied in IOR and HER. Recently, Wang et al.^[^ ^253^ ^]^ find the delocalized π electron system on carbon fiber paper (CFP) that induces promoted charge transfer with adsorbed I^−^ ions, thus resulting in desirable IOR catalytic activity of a low onset potential of only 0.54 V versus RHE. This result is approximate to theoretical value. The coupled system shows the cell voltage of only 0.59 V to reach 10 mA cm^−2^, which can reduce the energy consumption of 65% than conventional OWS, revealing higher energy conversion efficiency (Figure 10c,d).

##### Ascorbic Acid Oxidation

Ascorbic acid (AA) is a natural product that can be readily synthesized via biological fermentation.^[^ ^261^ ^]^ The oxidation product of dehydroascorbic acid have a wide applications such as cosmetic, medical, and pharmaceutical industries, etc.^[^ ^262^, ^263^ ^]^ In addition, the thermodynamic potential of AA oxidation reaction (AAOR) is 0.48 V versus RHE due to the reductive enediol group.^[^ ^264^ ^]^ The two‐electron transferred AAOR makes it energetically favorable than other organic substrates. In view of this, Cheng et al.^[^ ^254^ ^]^ propose the anodic AAOR and cathodic HER coupled system on Fe single atoms loaded on Ketjen black (Fe@KJ). The enol structure in AA is more reactive and the hydrogen atom is more easily to be activated that is different with the C–H and O–H bonds in alcohols or aldehydes. As a result, the AAOR exhibits a low overpotential of 12 mV to reach 10 mA cm^−2^, which is much lower than those of many biomass electrooxidations (urea, methanol, ethanol, glucose, glycerol, etc.) with higher overpotential of more than 600 mV (Figure 10e). The ^1^H NMR of electrolyte at 1‐h intervals is conducted to qualitatively analysis of AAOR products (Figure 10f). The ^1^H NMR before reaction shows three typical peaks at ≈4.92, ≈4.05, and ≈3.7 ppm, respectively are attributed to AA structure. With the time increase, three new peaks (1, 2, 3) are detected followed by the decrease intensity of AA structure. The yield rate of products can be reached to 87% at 0.75 V versus RHE for 4 h. To meet the industrial demand of utilizing AAOR‐coupled systems for hydrogen production, a two‐electrode coupled system using Fe@KJ and Pt mesh as anode and cathode electrodes, respectively, are constructed (Figure 10g). At 25 and 60 °C, such membrane‐free flow cell delivers 2 A cm^−2^ at the low cell voltages of only 1.5 and 1.1 V, respectively. The electricity consumption can be reduced to as low as 2.63 kWh m^−3^ H_2_ at 2 A cm^−2^, which is nearly half of the traditional OWS (≈5 kWh m^−3^ H_2_). This novel water electrolysis system expands the anodic alternative oxidation reactions to valuable enols to couple with OWS for low‐energy‐consumption hydrogen production and biomass upgrades.

##### Tetrahydroisoquinoline Oxidation

Dihydroisoquinolines (DHIQs) are kinds of value‐added biopharmaceutical intermediates for wide applications including antifungal, antitumor, vasodilation, and monoamine oxidase inhibition.^[^ ^266^, ^267^ ^]^ The potential synthesis procedures of DHIQs are dehydrogenation of tetrahydroisoquinolines (THIQs). However, the traditional methods including thermal or photocatalytic strategies usually involves environmentally harmful organic solvents. In addition, traditional strategies lead to the complete dehydrogenation products of isoquinolines (IQs), which results in higher energy consumption and decreased the value of the products.^[^ ^255^ ^]^ Therefore, it is more sustainable and atomically economical to accurately control the semidehydrogenation of THIQs to produce DHIQs through electrochemical oxidation. Taken together with the relatively low thermodynamic potential of THIQs semidehydrogenation (THIQs + 2OH^−^ → DHIQs + 2H_2_O + 2e^−^) than that of OER, the coupled of THIQs semi‐dehydrogenation and OWS can achieve low‐energy‐consumption hydrogen production and produced value‐added DHIQs chemicals.^[^ ^268^ ^]^ Zhang et al.^[^ ^269^ ^]^ firstly propose the selective semidehydrogenation of THIQs and coupled with OWS on a Ni_2_P electrocatalysts. In their contribution, the in situ formed Ni^II^/NiI^II^ redox species on Ni_2_P are active centers for precise semidehydrogenation of THIQs into DHIQs. In addition, the system can achieve the current density of 10 mA cm^−2^ with a reduced cell voltage of 300 mV compared with OWS, implying a great potential for the electrochemical production of both hydrogen and DHIQs chemicals.

**Figure 11:**
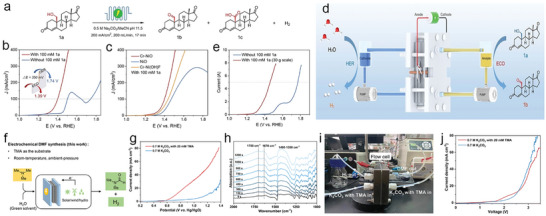
a) Electrooxidation reaction pathway of sterol oxidation. b) LSV curves of sterol oxidation with or without the addition of 100 mM sterol. c) LSV curves of different catalysts for sterol oxidation. d) Schematic illustration of coupled system for HER and sterol oxidation. e) LSV curves of coupled system with or without the addition of 100 mM sterol. Reproduced with permission.^[^ ^150^ ^]^ Copyright 2023. Wiley‐VCH. f) Concept of electrochemical synthesis of DMF through trimethylamine (TMA) oxidation. g) LSV curves on graphite plate in 0.7 M K_2_CO_3_ with or without the addition of 20 mM TMA. h) In situ IR absorption spectroscopy in 0.7 M K_2_CO_3_ with the addition of 20 mM TMA at 1.1 V versus RHE. i) Photograph of the flow reactor. j) LSV curves of the coupled system with or without the addition of 20 mM TMA. Reproduced with permission.^[^ ^265^ ^]^ Copyright 2023. Royal Society of Chemistry.

Recently, Zhao et al.^[^ ^255^ ^]^ further discuss the selectivity of the semidehydrogenation of THIQs on CoFe‐NiSe_2_ electrode. The onset potential of THIQs oxidation is evaluated by LSV tests (**Figure** **11**i). Compared with onset potential of OER (≈1.45 V vs RHE), the significant decrease of onset potential is observed with the addition of THIQs, indicating the electrooxidation of THIQs is thermodynamically more favorable than OER. In situ Raman and DFT calculations reveal that the selective oxidation of THIQs can be attributed to the in situ formed NiOOH on the CoFe‐NiSe_2_, and the dopant of Fe or Co controlled the first step of C–H cleavage rather than N–H cleavage, which is crucial for the THIQs oxidation to DHIQs so that the high‐energy‐consumption deep oxidation to produce IQs is avoided. Considering the low potential of THIQs oxidation, the OER is replaced by THIQs oxidation and coupled with HER and achieved low‐energy‐consumption hydrogen production (Figure 10j). DFT calculations further depict possible reaction path for the dehydrogenation of THIQs on CoFe‐NiSe_2_ (Figure 10k).

##### Sterol Oxidation

Steroid drugs are one of the most widely used chemical pharmaceuticals behind antibiotics for the treatment of rheumatic, endocrine, cardiovascular, skin and other diseases.^[^ ^270^ ^]^ The traditional methods for the oxidation of steroid usually involves hazardous chromic anhydride agents, which is harmful to environment and requires high energy consumption.^[^ ^271^ ^]^ Electrochemical pathway to oxidation of steroid is a green and facile strategy to synthesize complex steroid carbonyl products.^[^ ^272^ ^]^ However, the challenge remains due to the large steric hindrance of the complex structures and low water solubility of the steroid. In addition, considering the low thermodynamic potential of alcohol, the replacement of OER with steroid oxidation and coupled with OWS for hydrogen production is more energy efficient, but the low delivered current density and excessively long reaction time of the coupled system hindered the industrial application. In view of this, Wang et al.^[^ ^150^ ^]^ design a Cr‐doped NiO and Ni_3_N nanosheets on the graphite felt as bifunctional catalysts for HER and sterol (**1a**) oxidation (Figure 11a). Because the atypical electronic configuration (t^3^_2g_e^0^_g_) of Cr^3+^ can accelerate electron transfer and capture to provide favorable effect for Ni active sites. The three‐electrode LSV tests show that the potential reduced to 1.39 V versus RHE compared with OER (1.74 V, without the addition of **1a**) at 100 mA cm^−2^, indicating the thermodynamically more favorable of **1a** oxidation than OER (Figure 11b). The LSV tests for various catalysts (Cr‐NiO, NiO, and Cr‐Ni(OH)F) for **1a** oxidation shows the optimal activity on Cr‐NiO catalysts (Figure 11c). A two‐layer stacked electrochemical reactor is then built to scale up the electrocatalysts (Figure 11d). The Cr‐NiO and Cr‐Ni_3_N are used as anode and cathode electrodes, respectively. The cell voltage decreases significantly from 1.77 to 1.46 V at 5 A current, indicating the promising of energy‐saving hydrogen production and large‐scale value‐added medicine intermediates production of this novel coupled system (Figure 11e).

##### Waste Phenol Oxidation

Waste phenol is a significant threat to environment during the industrial manufacturing process because of its poor biodegradability. Traditional phenol treatment strategy usually involves chemical degradation and physical extraction, which may suffer serious secondary pollution and low efficiency. In addition, the exposure of phenol to air can inevitably generate by‐products of maleic acid, formic acid, and CO_2_. Electrochemical oxidation provides a cost‐effective and highly‐efficient way for value‐added chemical production such as benzoquinone. Benefiting from the successful integration of alcohol electrooxidation coupled OWS for H_2_ production, the electrochemical phenol oxidation reaction (EOP) coupled with OWS shows promising for environmental pollution issues treatment and low‐energy‐consumption H_2_ production. Based on this conception, Wen et al.^[^ ^273^ ^]^ propose EOP‐assisted OWS on highly‐efficient Ni_9_S_8_–Ni_15_O_16_ heterostructure supported on a nickel foam (NF) to construct a self‐supporting electrode, which is favorable for lowering the energy barrier of EOP. The Ni_9_S_8_ is preferred for C–H cleavage to yield C_6_H_4_O* intermediates, while the Ni_15_O_16_ component promotes the O* species formation from OH* discharge. As a result, the Ni_9_S_8_–Ni_15_O_16_/NF exhibits a low potential of 1.35 V versus RHE for EOP. The assemble of Ni_9_S_8_–Ni_15_O_16_/NF and commercial RuIr/Ti to construct EOP‐assisted OWS leads to an ultralow cell voltage of 0.6 V, to reach 10 mA cm^−2^, with a long‐term stability at 0.9 V for 12 days. The Faradaic efficiency of EOP to benzoquinone is as high as 93%. The coupling strategy provides a direction to advanced electrocatalyst design for highly‐efficient EOP and environmental pollution treatment. In addition, it offers a promising avenue for co‐production of hydrogen and benzoquinone chemicals.

##### Trimethylamine Oxidation

*N*,*N*‐dimethylformamide (DMF) is known to be used as solvent or crystallization agent in wide applications such as the production of plastic industry and acrylic fibers, agrochemicals, pharmaceuticals, adhesives, etc. Considering the continuously increase demand for DMF ($2.7 billion by 2027), it is urgent to develop green and facile method for the synthesis of DMF, as the traditional process involves the consumption of fossil‐based carbon monoxide and dimethylamine (DMA) under high temperature (50–200 °C) and pressure (0.5–11 MPa), where the DMA is synthesized by methanol amination. However, this process usually involves by‐products of monomethylamine (MMA) and trimethylamine (TMA) with much lower market value, which limits the overall energy efficiency for DMA production.

Recently, Duan et al.^[^ ^265^ ^]^ firstly propose a green and facile electrooxidation of TMA to produce DMF in an aqueous solution without any additives via 4e^−^ and 3H^+^ transferred process ((Me)_3_N → (Me)_2_N‐CHO) (Figure 11f). LSV curves conduct in the optimal condition (0.7 M K_2_CO_3_ with 20 mM TMA) of TMA oxidation and OER in pure 0.7 M K_2_CO_3_ shows a sharp increase in current density and decrease in working potential to 0.73 V versus Hg/HgO at 10 mA cm^−2^ with the addition of 20 mM TMA in 0.7 M K_2_CO_3_ compared with that in the absence of TMA (1.21 V vs Hg/HgO) (Figure 11g). Taken together with the reaction thermodynamics, the TMA oxidation shows a much low standard electrode potential of 0.32 V versus RHE, indicating a thermodynamically more favorable feature of TMA oxidation. In situ IR is conducted at 1.1 V versus Hg/HgO for 1200 s to investigate reaction mechanism of TMA oxidation (Figure 11h). The newly appeared peaks located at 1670 and 1755 cm^−1^ are attributed to the vibrational stretching of C═O in DMF and formaldehyde. The design of control experiments and EPR measurements further confirm their conjecture of TMA oxidation mechanism that the continuous oxidation of TMA to form amino radical cation, *α*‐amino carbon radical, iminium, and aminal intermediates derived the TMA oxidation to produce DMF. After that, they designs a two‐electrode flow reactor of anodic TMA oxidation and cathodic HER to meet the large demand of DMF in practice application (Figure 11i). Because of the thermodynamic favorable feature of TMA oxidation than OER, the onset cell voltage decreases to 2.0 V compared with conventional OWS (1.6 V) (Figure 11j). In addition, the OER dominates the reaction when the voltage is higher than 2.7 V, mainly due to the sluggish TMA diffusion in the flow reactor.

Apart from the TMA oxidation coupled OWS, Duan's group also introduce pioneer electrosynthesis of value‐added chemicals from biomass or plastic‐derived compounds.^[^ ^274^, ^275^, ^276^ ^]^ The comparison of recently reported other oxidation reaction and two‐electrode performance coupled with H_2_ production are listed in **Table** **4**.

**Table 4:** Comparison of recently reported other oxidation reaction and two‐electrode performance coupled with H_2_ production.

Substrate	Catalyst	Electrolyte	Oxidative potential (V vs RHE@10 mA cm^−2^)	Cell voltage (V@10 mA cm^−2^)	Ref.
**Sulfion**	CoS_2_@C/MXene/NF	1.0 M NaOH+1.0 M Na_2_S	≈0.4^a)^	0.68^b)^	[278]
**Sulfion**	*v*‐NiS_2_	0.1 M NaOH+50 mM Na_2_S	0.41	0.65	[222]
**Sulfion**	*n‐*Co_3_S_4_@NF	1.0 M NaOH+1.0 M Na_2_S	0.233^a)^	0.506^a)^	[233]
**Sulfion**	FeMo‐S/Ru	1.0 M NaOH+2.43 M Na_2_S	0.3^a)^	0.57^a)^	[59]
**Sulfion**	WS_2_ NSs	1.0 M NaOH+1.0 M Na_2_S	0.48	0.03	[279]
**Sulfion**	HEA‐Mo_2_C/HPC	1.0 M NaOH+1.0 M Na_2_S	0.382^a)^	0.83^a)^	[280]
**Sulfion**	Co_3_S_4_	1.0 M NaOH+1.0 M Na_2_S	0.262^a)^	0.496^a)^	[239]
**Sulfion**	NiSe/NF	1.0 M NaOH+1.0 M Na_2_S	0.49^a)^	−	[281]
**Sulfur dioxide**	A‐CP	0.5 M H_2_SO_4_+0.1 M FeCl_3_+ SO_2_ inflow	≈0.95	0.97^a)^	[282]
**Benzylamine**	Vo‐CuO/CF	1.0 M KOH+25 mM BA	≈0.52	−	[60]
**Cyclohexenylethylamine**	NiO/NC	1.0 M KOH+25 mM Amines	1.38	−	[251]
**Benzylamine**	Fe‐Ni_3_S_2_	1.0 M KOH+10 mM BA	1.36	−	[245]
**Butylamine**	CoSe_2_/Ni−SVs	1.0 M KOH+20 mM BuA	1.52	−	[223]
**Benzylamine**	CoSe_2_/Ni−SVs	1.0 M KOH+ BA	≈1.33	1.37	[223]
**Cyclohexane methylamin**	CoSe_2_/Ni−SVs	1.0 M KOH+ CM	≈1.34	−	[223]
**NaI**	CFP	0.1 M HClO_4_+1.0 M NaI	0.54	0.59	[253]
**NaI**	RuTiO	0.1 M HClO_4_+1.0 M NaI	≈1.0	1.09	[260]
**KI**	Ni‐Co(OH)_2_ NSAs	1.0 M KOH+0.33 M KI	1.30^c)^	1.34	[259]
**Ascorbic acid**	Fe@KJ	1.0 M Na_2_SO_4_+1.0 M AA	0.492	1.5^d)^	[254]
**Tetrahydroisoquinolines**	CoFe‐NiSe_2_	1.0 M KOH+10 mM THIQs	1.31	−	[255]
**Sterol**	Cr‐Ni_3_N/GF	1.0 M Na_2_CO_3_+100 mM Sterol	1.39^a)^	1.46^e)^	[150]
**Phenol**	Ni_9_S_8_‐Ni_15_O_16_/NF	1.0 M KOH+5 mM Phenol	1.30	0.95	[273]
**Trimethylamine**	Graphite flake	0.7 M K_2_CO_3_+20 mM TMA	0.75^f)^	≈2.4	[265]

^a)^ Current density (100 mA cm^−2^);

^b)^ Current density (200 mA cm^−2^);

^c)^ Current density (20 mA cm^−2^);

^d)^ Current density (2 A cm^−2^);

^e)^ Current (5 A);

^f)^ Versus Ag/AgCl.

#### 2.2.4 Bipolar Hydrogen Production

Although the concept of coupled system for energy‐efficient hydrogen production with organic oxidation reactions (e.g., furfural, alcohol, etc.) have shown much promise and progress, these conventional electrolysers for hydrogen production still require high cell voltage input (>1 V), resulting in higher energy consumption. In view of this, Wang et al.^[^ ^110^ ^]^ first propose a conception of hydrogen production from anode and cathode from low‐potential aldehyde oxidation coupled OWS coupled system. In their contribution, the H atoms from aldehyde are released as H_2_ rather than less valued H_2_O to meet the atom economy principle in green chemistry. Otherwise, a higher working potential (>1 V vs RHE) will be applied to oxidize adsorbed H* from C–H cleavage of aldehyde to produce H_2_O via high‐energy‐consumption Volmer step (R–CHO + 3OH^−^ ⇔ R–COO− + 2H_2_O + 2e^−^). The low‐potential aldehyde oxidation reaction is crucial to achieve the selective C–H bond cleavage at lower potential (0.1–0.4 V vs RHE) with the production of 5‐hydroxymethyl‐2‐furancarboxylic acid (HMFCA) and H_2_ on the Cu electrode via the reaction: R–CHO + OH^−^ = R–COO^−^ + H_2_. As a result, the integrated two‐electrode system using Cu and Pt/C as anode and cathode respectively shows ultralow onset cell voltage of below 0.1 V, and only 0.27 V at target current density of 100 mA cm^−2^. The corresponding electricity input of the bipolar hydrogen production system is as low as ≈0.35 kWh cm^−3^ of H_2_ at 100 mA cm^−2^, which is one‐fourteen of the conventional OWS (≈5.0 kWh  m^−3^ of H_2_) due to the unique one electron transferred reaction and ultralow applied cell voltage for hydrogen production. Afterward, they integrate the low‐furfural oxidation, hydrogen production, and electricity output in one coupled system instead of the conventional coupled system that requires electricity input.^[^ ^89^ ^]^ In their another contribution, small molecule aldehydes of formaldehyde (HCHO) with higher hydrogen content is introduced in bipolar hydrogen production system.^[^ ^283^ ^]^ The partially reduced CuO on Cu foam is synthesized for formaldehyde oxidation reaction (FOR). Several in situ characterizations such as XAS, ATR‐FTIR combined with DFT calculations revealed that Cu^0^ sites promote the reaction energy of HOCH_2_O* + HO*, while Cu^+^ sites are more favorable to cleavage C–H bond. The in situ DEMS confirms that the H of H_2_ is all derived from HCHO. Recently, they further design a mixed‐valenced Cu catalysts (MV Cu) for enhanced low‐furfural oxidation.^[^ ^284^ ^]^ They propose that the poor activity for the low‐furfural oxidation of Cu foam is attributed to the weak adsorption ability of furfural on Cu^0^, while the Cu_2_O provides insufficient adsorption sites for furfural since the Cu^+^ is existed in the form of Cu(OH)_ads_. Thus, the regulation of valence of Cu is key for promoting low‐furfural oxidation. The constructed dual‐side H_2_ production device shows a low cell voltage of 0.3 V with an electricity input of 0.24 kWh mH_2_^−3^, which is far lower than that of conventional OWS.

**Figure 12:**
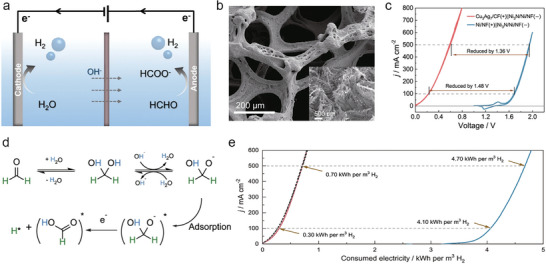
a) Concept illustration of electrocatalytic dual hydrogenation design. b) SEM image of Cu_3_Ag_7_/CF. c) CV curves of the two‐electrode with or without the addition of 0.6 M HCHO. d) Possible mechanism of HCHO oxidation. e) Electricity consumption for H_2_ production on formaldehyde (red) or paraformaldehyde (black) oxidation coupled OWS and the traditional OWS. Reproduced with permission.^[^ ^277^ ^]^ Copyright 2023. Springer Nature.

Following the conception of low‐potential aldehyde oxidation and bipolar hydrogen production Sun et al.^[^ ^277^ ^]^ introduce the hydrogen storage molecule of HCHO in OWS to achieve bipolar hydrogen production at both anode and cathode due to the low thermodynamic potential of partial oxidation of HCHO to form formate accompanied by H_2_ release (HCHO + 2OH^−^ → HCOO^−^ + 1/2H_2_ + H_2_O + e^−^, *E* = –0.22 V vs RHE) (**Figure** **12**a). This bipolar hydrogen production conception can avoid the tremendous disparity of anodic products and large‐scale demand of H_2_ fuel. In their contribution, a Cu_3_Ag_7_ catalyst is electrodeposited on the 3D porous copper foam (Cu_3_Ag_7_/CF) as anode electrode (Figure 12b). A two‐electrode electrolyzer with Cu_3_Ag_7_/CF and Ni_3_N/Ni/NF used as anode and cathode electrodes are built. CV curves show that the cell voltages of 1.48 and 1.36 V can be reduced with the addition of 0.6 M HCHO in 1.0 M KOH (Figure 12c). The proposed mechanism of HCHO oxidation is that the H_2_C(OH)O anion is formed by the hydration and deprotonation of HCHO. The H_2_C(OH)O is then adsorbed onto the catalysts (H_2_C(OH)O*) and then undergoing dehydrogenation via C–H cleavage to obtain HCOOH* and H* (Figure 12d). Theoretical calculations reveal that the H_2_C(OH)O* intermediates are more favorably adsorbed on Cu_3_Ag_7_ alloy, which can also promote the C–H cleavage and H* recombination to form H_2_. This bipolar hydrogen production system also expands the substrate scope to paraformaldehyde oxidation. CV curves show that the much lower potentials of 0.13 and 0.36 V versus RHE are delivered than those of conventional OWS to reach 100 and 500 mA cm^−2^, respectively. The electricity input is as low as 0.30 kWh m^−3^ H_2_ at 100 mA cm^−2^ (Figure 12e). In another report, they proposed an electrocatalytic dual hydrogenation strategy on Pd membrane to avoid market‐size mismatch of anode and cathode products in the traditional coupled system and achieved Faradaic efficiency of formaldehyde to formate approaching 200%.^[^ ^285^ ^]^ The comparison of recently reported low‐potential oxidation reaction for bipolar hydrogen production are listed in **Table** **5**. From the above pioneer works for designing energy‐saving bipolar hydrogen production systems, it can be estimated that the conception of coupled water electrolysis systems have a great potential for future practical application on a large scale.

**Table 5:** Comparison of recently reported low‐potential oxidation reaction for bipolar hydrogen production.

Substrate	Catalyst	Electrolyte	Oxidative potential (V vs RHE@10 mA cm^−2^)	Cell voltage (V@100 mA cm^−2^)	Electricity input (kWh m^−3^ H_2_@100 mA cm^−2^)	Ref.
**HMF**	Cu	1.0 M KOH+100 mM HMF	0.12^a)^	0.27	0.35	[110]
**Furfural**	CuAg_glv_/Cu	1.0 M KOH+200 mM 2‐FA	≈0.40	0.21	−	[286]
**Formaldehyde**	CuAg/CF	1.0 M KOH+0.6 M HCHO	0.40	0.22	0.30	[277]
**Formaldehyde**	Pd membrane	1.0 M KOH+0.6 M HCHO	−	1.0^b)^	−	[285]
**Formaldehyde**	Cu*_x_* O@CF	1.0 M KOH+0.1 M HCHO	≈–0.07	−	−	[283]
**Formaldehyde**	CF@Cu‐NS	1.0 M KOH+50 mM HCHO	≈–0.05	−	−	[162]
**Furfural**	MV Cu	1.0 M KOH+50 mM FF	0.25	0.18	0.24^c)^	[284]
**Formaldehyde**	PdCu	1.0 M KOH+200 mM HCHO	≈0.3	0.13^a)^	0.13	[287]
**1,3‐Propandiol**	PdBi/NF	1.5 M KOH+50 mM FF	≈0.6^a)^	0.86^d)^	−	[288]
**Furfural**	RuCu NW	1.0 M KOH+50 mM FF	0.37^e)^	0.43	−	[49]
**1,6‐Hexanediamine**	NiNF	1.0 M KOH+25 mM Amine	≈1.40	−	−	[289]

^a)^ Current density (100 mA cm^−2^);

^b)^ Current density (15 mA cm^−2^);

^c)^ Applied voltage (0.3 V);

^d)^ Current density (20 mA cm^−2^);

^e)^ Current density (150 mA cm^−2^)

## 3 Conclusions

**Figure 13:**
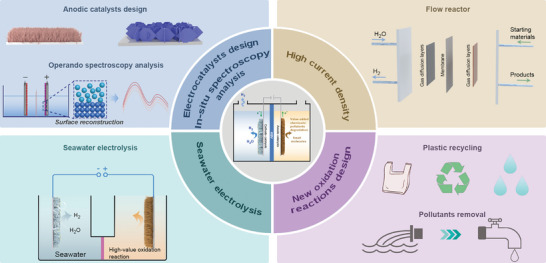
Illustration of future development of coupled water electrolysis.

This review discusses the recent advances in coupling water‐splitting hydrogen generation with value‐added electrochemical transformation of small molecules, such as aldehydes, alcohols, amines, and H_2_S, to value‐added chemicals or electrochemical degradation of pollutants (hydrazine, urea). In addition, these oxidation reactions provide a favorably thermodynamic driving force than OER to achieve hydrogen production with a low cell voltage, and hence, low energy consumption. In view of the rapid development of this coupling strategy, it is feasible to couple OWS with many other new molecules, such as tetrazoles, furazans, iodide, quinolines, ascorbic acid, sterol and trimethylamine, used in various fields (pharmaceuticals, agrochemicals, etc.), to generate a range of value‐added chemicals. Moreover, considering the market‐size mismatch of large‐scale demand of H_2_ fuel and anode products, a new mechanism of bipolar hydrogen production at both anode and cathode is proposed to overcome this drawback brought by conventional coupled systems. Such electrochemically coupled systems also benefit from the increasing availability of low‐cost renewable electricity, to reduce CO_2_ emissions in fuel and chemical productions. Despite the significant advances have been made of such a coupled approach, there are still several challenges to overcome as it is just at an infant stage. The fundamental research should be put forward to enhance the development and practical application of coupled system (**Figure** **13**).

1. Electrocatalysts play a crucial role in the high selectivity and yield rate of the anodic small molecules oxidation. However, many reports mainly emphasize on the design of highly‐efficient cathodic HER catalysts, while the investigation of catalyst electrodes for anode is overlooked since the anodic activity for small molecules oxidation are still much higher than thermodynamic values. There is more space to develop highly‐efficient anodic catalysts for promoting overall performance of coupled system. It is noted that the majority of the electrocatalysts for SMOs are based on Fe, Co, Ni, Mn, etc., but these transition metal‐based electrocatalysts are also active for competitive OER, leading to a low Faradaic efficiency, conversion rate, selectivity, and yield of a target value‐added oxidative product. The electrochemical window for high‐yield products becomes narrow on paired architectures. It is appealing to fundamentally comprehend and control the differences between OER and SMO, such as developing OER inert copper‐based electrocatalysts via several strategies including active sites regulation (defect engineering, alloying, doping, etc.) and morphology regulation (3D self‐standing nanosheets, nanowires, nanorod, etc.) so as to improve the intrinsic activity of electrocatalysts. In addition, several small molecules contain different functional groups. The understanding of the relationship between specific functional groups and active sites of electrocatalysts is required to accurately design the electrocatalysts for target SMOs. Furthermore, the design of anodic and cathodic electrocatalysts separately is not ideal since the anode catalysts may entail dependencies on the cathode catalysts. Therefore, developing bifunctional electrocatalysts toward SMOs and HER are warranted. On the other hand, nonprecious electrocatalysts usually suffer from poor stability, especially under alkaline conditions. The incorporation of second metal such as Ag to induce surface reconstruction and stabilize transition‐metal‐based electrocatalysts substrates is feasible since Ag is considered to be more durable and active to catalyze SMOs reaction. The surface structure of anodic catalysts is usually reconstructed during continuously operation, the main active components and reaction may be ceaselessly changed, leading to the dilemma of detecting real active center and investigation of adsorption/desorption capacity on active sites. Therefore, the cutting‐edge *operando* spectroscopy analysis such as in situ Raman, FTIR, XANES, etc., can be employed to investigate the nature of structural evolution and adsorption/desorption features of intermediates on the catalysts surface.
2. The requirement of higher current density is necessary for large‐scale application. Despite the advanced catalysts have been designed for high‐efficiency activity for coupled water electrolysis systems, the activity, stability, and selectivity, etc., are usually tested under low current density (<100 mA cm^−2^), which is unsatisfactory for industrial application (>500 mA cm^−2^). Therefore, the design of highly‐efficient self‐supporting electrocatalysts with robust performance for high‐current‐density and long‐term stability is desirable. In addition, the ingenious flow reactor can overcome the drawbacks brought by conventional H‐type electrolyzer such as high resistance, low current density, high cell voltage, and low Faradic Efficiency. In addition, the issues of selectivity of ion‐exchange membranes should be considered since the organic small molecule substrates may be crossed to counter electrode and decrease the product yield. More importantly, the shuttle of substrates may affect the membrane and catalysts electrode, it is going to get worse when operating under high concentration especially in flow reactor. Therefore, attention should be paid to the compatibility of different reaction systems, substrate supply methods, and water management with the selection of suitable ion‐exchange membranes. The design of flow reactor has more potential for practical application, while the cost of starting materials, downstream separation, purification issues, and economics (e.g., overall energy cost), etc., should be taken further consideration.
3. Direct seawater electrolysis shows promising for sustainable hydrogen production because the seawater takes up 96.5% of the water on the earth, however, few efforts have been devoted on this, let alone the coupled seawater electrolysis. The main challenge is that the ClOR as the competitive reaction may reduce the Faradic efficiency of the cell and produce toxic chlorine. In addition, the strong binding strength of Cl^−^ with active sites may poison the active centers for further reaction, which will expedite the corrosion of catalysts and reduces the long‐term stability of catalysts. The above‐mentioned problems may be more serious under high current density (>200 mA cm^−2^). As for the coupled seawater electrolysis, another issue should be concerned that the pH changes and ionic impurities in seawater electrolytes may induce more side reactions and reduce selectivity and yield rate of the target cathode and anode products. The design of stable seawater electrocatalysts with no corrosive effect on the electrode are required for coupled seawater electrolysis. Moreover, the in‐depth understanding of the mechanism in virtue of in situ techniques (Raman, FTIR, XRD, etc.) during seawater electrolysis on the catalysts can indicate the direction to elaborately construct high‐performance coupled seawater electrolysis system.
4. Exploration of pH‐independent nonprecious metal electrocatalysts is crucial to accommodate various pH conditions such as alkaline, acidic, and neutral environment. Proton exchange membranes (PEMs) water electrolysis can provide abundant proton environment for decreasing the ohmic resistance and promote Volmer step to contribute hydrogen production. However, the corrosive acidic electrolyte required noble catalysts for stable catalysis. While nonprecious are more appealing in alkaline anion exchange membranes (AEMs) water electrolysis, the sluggish Volmer kinetic of HER and harsh condition in alkaline medium may passivate catalysts. Therefore, electrocatalysts are limited to work in one particular environment that is unconducive for large‐scale applications. Although considerable progress has been made on highly‐efficient electrocatalysts in a wide pH condition, it has to be recognized that there is still a large gap between laboratory‐scale operation and industrial production. For instance, the majority of current experiments are evaluated in relative mild electrolytes (0.5 M H_2_SO_4_, 1.0 M KOH, and 1.0 M PBS) at room temperature, while the operation conditions in real water electrolysis equipment usually requires more condensed electrolytes such as 6–8 M KOH solutions at an evaluated temperature (50–90 °C). This requires exploring robust electrocatalysts to well endure corrosion and ion poisoning to approach practical devices.
5. Product separation is another essential operational step during chemical production in practice, yet most of the previous reports on coupled water electrolysis mainly focus on highly‐efficient electrocatalyst design and value‐added product generation, the issues of “postreaction,” such as product separation and economic viability of the system, are often overlooked. For example, alkaline (e.g., 1.0 M KOH) media are always employed in most coupled water electrolysis systems, such as HMFOR and alcohol oxidations, due to its high performance, but the high solubility of products in such conditions require extra acidization process to extract the final products. The product such as FDCA is nearly insoluble in acidic water (0.1 g/100 mL water). However, such acidic process adds cost in industrial production. In addition, the real products in alkaline media are potassium salts of acetates, formate, and lactates. These metal salts are of lower market demand and less‐valued, relative to their carboxylic acid counterparts. Therefore, exploring highly‐efficient acidic or even more‐mild neutral reaction conditions is desirable to reduce the separation cost and corrosiveness to equipment because the coveted neutral or near‐neutral conditions can alleviate the side reactions (e.g., polymerization of aldol condensation during HMFOR) and evade extra neutralization steps in products separation process. Additionally, the separation of soluble products in a low concentration is of high cost, it is highly appealing to extract insoluble products at a relatively high concentration (>500 mM). Moreover, the economic viability of the whole system should be considered. The merits of OER‐substituted water electrolysis system are to reduce the energy input for H_2_ production and value‐added chemicals or efficient pollutant degradation, hence the whole cost of the water electrolysis can be reduced. Therefore, assessmentof real economic feasibility should be evaluated. Some factors that influence the profitability, such as the cost of raw materials, device components, electrocatalysts, electricity, electrolytes, and products separation, should be considered. In view of the above, the techno‐economic analysis, such as (TEA) of minimum selling price (MSP) and return on investment (ROI), should be carried out in future study of coupled water electrolysis systems, to better assess their economic feasibility for practical applications.
6. From the perspective of carbon neutral, green, and sustainable conceptions, the development of new alternative oxidation reactions are necessary such as biomass upgrading, waste streams, plastic recycling, or pollutants removal, etc. The assemble of the above reactions with HER can realize the production of more kinds of value‐added chemicals or purification of water from industrial wastes, as well as hydrogen production under lower electricity input. In addition, the “green electricity” obtained from renewable resources such as solar, wind, etc., are highly praised instead of fossil energy.

Taken together, this review highlights the development of coupled green electrooxidation of small molecules with OWS for improving the energy efficiency of hydrogen production, and provides a green pathway for value‐added chemicals production or pollutant degradation. We believe that through the further development of highly‐efficient and robust electrocatalysts design, in‐depth mechanism investigation, and large‐scale tests of the coupled water electrolysis, it is appealing to put this technology forward to the practical coupled chemical manufacturing of clean hydrogen and chemicals.

## Conflict of Interest

The authors declare no conflict of interest.
